# “Molecular Masks” for ACE2 to Effectively and Safely Block SARS-CoV-2 Virus Entry

**DOI:** 10.3390/ijms22168963

**Published:** 2021-08-20

**Authors:** Satya Prakash Shukla, Kwang Bog Cho, Vineeta Rustagi, Xiang Gao, Xinping Fu, Shaun Xiaoliu Zhang, Bin Guo, D. Gomika Udugamasooriya

**Affiliations:** 1 Department of Pharmacological & Pharmaceutical Sciences, University of Houston, 4849 Calhoun Rd, Houston, TX 77204-5037, USA; spshukla@central.uh.edu (S.P.S.); kcho7@central.uh.edu (K.B.C.); vrustagi@uh.edu (V.R.); xgao23@central.uh.edu (X.G.); 2 Department of Biology and Biochemistry, University of Houston, 3455 Cullen Blvd, Houston, TX 77204-5037, USA; xfu3@central.uh.edu (X.F.); xzhang5@central.uh.edu (S.X.Z.); 3 MD Anderson Cancer Center, Department of Cancer Systems Imaging, 1881 East Road, Houston, TX 77030-4009, USA

**Keywords:** SARS-CoV-2, ACE2 receptor, peptoids

## Abstract

Coronavirus Disease 2019 (COVID-19) remains a global health crisis, despite the development and success of vaccines in certain countries. Severe acute respiratory syndrome coronavirus 2 (SARS-CoV-2), the virus that causes COVID-19, uses its spike protein to bind to the human cell surface receptor angiotensin-converting enzyme 2 (ACE2), which allows the virus to enter the human body. Using our unique cell screening technology, we identified two ACE2-binding peptoid compounds and developed dimeric derivatives (ACE2P1D1 and ACE2P2D1) that effectively blocked spike protein-ACE2 interaction, resulting in the inhibition of SARS-CoV-2 pseudovirus entry into human cells. ACE2P1D1 and ACE2P2D1 also blocked infection by a D614G mutant pseudovirus. More importantly, these compounds do not decrease ACE2 expression nor its enzyme activity (which is important in normal blood pressure regulation), suggesting safe applicability in humans

## 1. Introduction

Since the start of the COVID-19 pandemic, vaccine development has represented the main research and development effort to combat this deadly disease. To date, multiple vaccines have been approved by the United States Food and Drug Administration (FDA) for emergency use, and they are showing an initial success in controlling the pandemic in certain countries. Yet, the pandemic is far from over, as new strains of the SARS-CoV-2 virus with different new mutations are emerging from the continued outbreaks in various countries on different continents. The potency of current vaccines against these novel strains and the newer strains appearing in the future are not known. Reports of people who are fully vaccinated and yet are still being infected with the SARS-CoV-2 virus raise the concern over the efficacy of vaccine protection, especially with the new Delta and Lambda variants. Therefore, alternative approaches are highly warranted to find lasting solutions for both prevention and treatment of COVID-19. This is particularly important for the world to return to normal pre-pandemic life and work.

**Figure 1:**
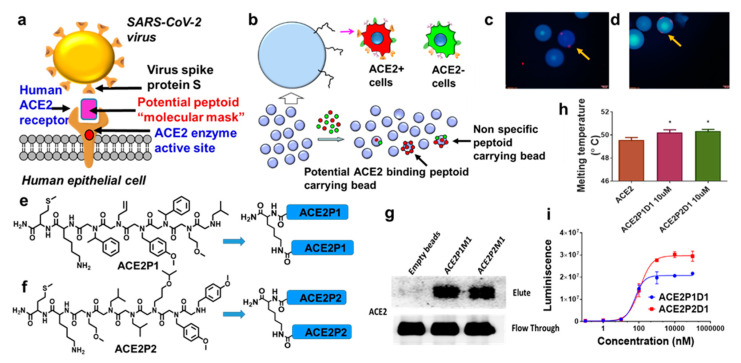
OBTC peptoid screen identifies two potential human ACE2 binding peptoids. (**a**) Schematic representation of SARS-CoV-2 virus spike protein S binding to human ACE2 receptor and the idea of developing ACE2 receptor binding peptoids as “molecular masks”. (**b**) Outline of the OBTC assay. Two identical cell groups that defer only by ACE2 receptor expression were exposed to 50,000 TentGel resin beads that display peptoids in one-bead one-compound library format. ACE2+ (positive) MCF-7 cell group was stained in red, and AC2- (knocked down) MCF-7 cell group was stained in green, mixed 1:1 and exposed to the peptoid library. A bead bound only with red stained cells indicates having the peptoid ligand that selectively bound to ACE2 receptor over all other molecules found on MCF-7 cell surface. Beads bound with both red and green cells were discarded as non-specific peptoid carrying beads. (**c**,**d**) Fluorescence microscopic images of beads bound with only red stained cells (only two such beads were found, as indicated by the arrows). (**e**,**f**) Chemical structures of peptoids found on those 2 beads, named as ACE2P1 and ACE2P2, respectively. The optimized simple dimerized structures ACE2P1D1 and ACE2P2D1 are shown after the arrows. (**g**) TentaGel beads carrying ACE2P1 and ACE2P2 peptoids or empty beads (as control) were incubated with recombinant ACE2 protein. The beads were washed thoroughly, and protein was eluted and analyzed by Western blot. Blots were cropped from different parts of the same gel. (**h**) Recombinant ACE2 protein (5 µg) was incubated with 10 µM ACE2P1D1 or ACE2P2D1 (dimer form of the peptoids). Thermal shift assay was performed using a QuantStudio 3 Real-Time PCR System. Each bar represents the mean + standard deviation (*n* = 3/group), * *p* < 0.05. (**i**) ELISA-like quantitative ACE2-peptoid binding assay was performed with coating 96-well plates with recombinant human ACE2 treatment with increasing concentrations of peptoids and probing bound peptoids via luminescence. The data analysis indicates dissociation constant (Kd) values of 60 and 110 nM. The error bars represent the standard deviation between duplicates.

The SARS-CoV-2 virus that is causing the current global health crisis belongs to the SARS family of coronaviruses [1,2]. These coronaviruses (including SARS-CoV, SARS-CoV-2, MERS-CoV, and others) use their spike proteins to bind to the human ACE2 protein, which enables virus entry into human cells [3,4,5,6]. It has been known that Transmembrane Serine Protease 2 (TMPRSS2) receptor and other proteases are also involved in SARS-CoV-2 cell entry [7]. However, TMPRSS2 acts after the virus binds to ACE2 [3]. Without spike-ACE2 interaction, TMPRSS2 cannot allow viral entry. This is evidenced by the failure of the virus to infect ACE2 negative HeLa cells [5], which express TMPRSS2 [8]. In the meantime, SARS-CoV-2 can infect 293T cells expressing ACE2 but not TMPRSS2 [3]. During SARS-CoV-2 infection, the spike protein undergoes conformational changes involving several protein domains, such as the receptor binding domain (RBD), N-terminal domain (NTD), and S1 and S2 subunits. This changes the RBD from down to up conformation and prepares the virus for binding to the ACE2 receptor and the fusion of the viral and cell membranes and the final release of the viral RNA into the cytoplasm [9]. The mechanical stability of the RBD of SARS-CoV-2 is stiffer (greater by 50 pN) compared to SARS-CoV; thus, it can withstand Brownian and cellular forces and yet maintains close contact while priming of the spike protein by TMPRSS2 [10]. The structure of the SARS-CoV-2 RBD-ACE2 complex has been solved using X-ray crystallography [11]. The overall binding mode of the SARS-CoV-2 RBD to ACE2 is nearly identical to that observed in the previously determined structure of the SARS-CoV RBD-ACE2 complex. Therefore, developing molecules that can bind to ACE2 and block this interphase between viral spike protein and ACE2 can be useful as molecular blocking agents to prevent virus entry (Figure 1a). As the family of coronaviruses has been studied for about two decades, ACE2 binding agents, including small molecules [12,13], peptides [14,15,16,17], and antibodies [18] have been reported to block virus spike-ACE2 interaction [19]. However, no ACE2-targeted compound has been approved as an antiviral drug to be used in the clinic. This may be the result of serious concerns over the inhibition of ACE2 enzymatic activity by targeting ACE2, which will interfere with the normal function of ACE2, such as blood pressure regulation [20].

The conventional ligand or drug-lead discovery tools such as structure-based or high throughput screening (HTS) often identify molecules that bind to the active site of the targeted protein. These methods are not successful in ACE2-targeted drug discovery to block viral infection due to two reasons. First, the drug should preferably bind to the interphase where the virus spike protein binds, which is now known to be away from the ACE2 enzymatic active site [21]. Second, the drug should not affect the physiological functions of ACE2; otherwise, the drug will have serious side effects (such as affecting blood pressure regulation). Almost all ACE2-targeted small molecules reported thus far seem to bind to the ACE2 active site [12,13], which may cause major health problems and disqualify them to be used as molecular masks. Therefore, novel approaches are needed to find synthetic molecules that bind to the ACE2 on the virus spike protein binding surface and are capable of blocking the spike protein-ACE2 interaction yet having no effect on ACE2 enzymatic activity. Recent computational studies have reported certain peptides binding to this interphase that can block the virus spike protein-ACE2 interaction [22,23,24]. While certain peptides [14,15,16,17] and antibodies [18,19] reported seeming to bind to this interphase area, none of them has been developed into actual drugs thus far, indicating that avoiding ACE2-related side effects is a daunting task.

To overcome these hurdles in ACE2-targeted drug discovery, we have applied a unique combinatorial HTS technology that we developed previously to screen for new synthetic ACE2-binding ligands. Our on-bead two-color (OBTC) cell screening technology (Figure 1b) identifies synthetic ligands based on target binding instead of the conventional function-based screens [25,26,27]. OBTC compares two cell groups that differ only by the presence or absence of the target in real time and identifies ligands that only bind to the target and not any other common cell surface molecules. Therefore, this method guarantees the high selectivity of the identified ligand for the target. In addition, this assay allows for identifying ligands binding to anywhere on the receptor surface, preferably the open areas, making it possible to identify ligands that hit the spike protein binding interphase on ACE2. Most importantly, since we expose the ACE2 receptor under its natural expression condition on a live cell (as the whole system) to a library of synthetic ligands (displayed on beads) in this OBTC assay, the potential binding ligands may prefer to bind to the tip of the ACE2 receptor, an area where the virus also selected as its binding site in the viral evolution [28,29]. We chose peptoids as the synthetic ligands to target ACE2 for several reasons. Peptoids closely resemble the natural peptides [30] and are protease-resistant, serum stable, and highly tissue-permeable [31,32,33,34,35]. Their on-bead synthesis is straightforward [34] and each coupling step can be completed in 30 seconds microwave pulses [36]. We and others have demonstrated that peptoids are rich sources of protein-binding ligands [25,26,27,31,35,37,38,39] and are non-immunogenic in mice [40]. Peptoid modifications are straightforward and have moderate clearance [41]. Therefore, we believe peptoids can serve as a drug-like, biologically amenable, and economical class of molecules [35,42,43,44,45] that may be developed into effective therapeutics or formulations of nasal sprays or eye drops.

## 2. Results

### 2.1. OBTC Screening Identified Two Novel ACE2-Binding Peptoids

We have developed a simple monomeric peptoid library using a standard split-pool synthesis procedure (Supplementary Figure S1) [46] for the OBTC screening of ACE2-binding compounds. We selected MCF-7 cell line as our model system as it has been reported to express ACE2 protein. We used siRNA to effectively knock down ACE2 in MCF-7 cells and used these cell group as ACE2 negative group in the OBTC assay (Supplementary Figure S2). As per our original assay protocol, we first stained ACE2 positive MCF-7 cell group in red (quantum dot 655) and the ACE2 negative (siRNA knockdown) MCF-7 cell group in green (quantum dot 565), mixed the two groups at 1:1 ratio and exposed to 50,000 beads of the above mentioned peptoid library. After equilibration, unbound cells were washed out and the beads were screened under a fluorescence microscope. We found two beads that bound only to red stained cells, indicating that the peptoids displayed on these beads bind only to ACE2 and not to any other cell surface molecules present on both cell groups (Figure 1c,d). These beads were picked and processed to clean up by removing proteins and cell debris. The peptoids on the two beads were cleaved off the resin and subjected to MALDI-MS/MS sequencing, which identified the two peptoid sequences. We named them as ACE2P1 and ACE2P2 (Figure 1e,f). We also developed simple dimeric versions of both peptoids, as multimers of such ligands are known to improve binding affinities for targeting cell surface receptors [25,27]. A central lysine residue was used as the linker between two monomeric units (Figure 1e,f) and the fully on-bead synthesis protocol was utilized to synthesized these dimers, as we previously published [47].

### 2.2. Direct Binding of Peptoids ACE2P1D1 and ACE2P2D1 to ACE2

We first validated the OBTC screening results by resynthesizing ACE2P1 and ACE2P2 on tentagel beads and incubated them with red cells (ACE2 positive) and green cells (ACE2 negative) separately as well as 1:1 mixture of these cells. We found red cells predominantly bound with those beads while green cells had no binding (Supplementary Figure S3). Next, we incubated recombinant ACE2 protein with the ACE2P1 or ACE2P2 conjugated tentagel beads and performed in vitro pull-down assay. As shown in Figure 1g, ACE2P1 and ACE2P2 beads successfully pulled down ACE2 protein from the solution. As a control, the empty tentagel beads did not pull down ACE2 protein. To confirm a direct interaction between the peptoids and ACE2, we performed a thermal shift assay using recombinant ACE2 protein. The Applied Biosystems™ Protein Thermal Shift™ assay measures protein thermal stability using a fluorescent protein-binding dye. The Protein Thermal Shift dye does not fluoresce in aqueous solutions but fluoresces in nonpolar environments. The protein is mixed with the dye and heated; as it unfolds or melts, hydrophobic parts of the protein are exposed and bind to the dye, resulting in fluorescence emission detected by the qPCR system. Binding of a ligand to the protein changes the stability of the protein, resulting in a change in fluorescence intensity. As shown in Figure 1h, when recombinant ACE2 was mixed with ACE2P1D1 or ACE2P2D1, increases in protein melting temperature were observed, indicating a direct interaction between these compounds and ACE2. Finally, we performed a previously published ELISA-like quantitative binding assay to quantify the peptoid-ACE2 binding event [27]. The recombinant His-tagged ACE2 protein was attached to the Nickel-coated 96-well plate. Biotinylated ACE2P1D1 and biotinylated ACE2P2D1 were introduced with varying concentrations. After washing, the remaining compounds were probed with streptavidin-HRP system followed by detecting luminescence signals using a spectrophotometer. We found that ACE2P1D1 and ACE2P2D1 bind to ACE2 protein with Kd values of 60 and 110 nM (Figure 1i).

### 2.3. ACE2P1D1 and ACE2P2D1 Blocked SARS-CoV-2 S Protein Binding to ACE2

**Figure 2:**
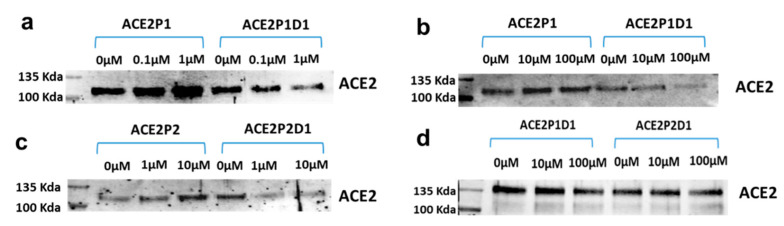
ACE2P1 and ACE2P2 dimers blocked the binding of spike protein to ACE2. (**a**–**c**) Recombinant ACE2 (0.1 µg/sample) and recombinant GST-tagged SARS-CoV2 spike proteins (0.1 µg/sample) were mixed together. ACE2P1 and ACE2P2 monomers or dimers (D1) were premixed with ACE2 for 1 h before the mixture was applied to the GST pull-down assay to determine if these compounds can block the interaction between ACE2 and spike protein. ACE2 in the pull-down assay was detected by western blot. (**d**) The interaction of recombinant ACE2 and D614G spike proteins (0.1 µg/sample) was determined by pull-down assay in the presence or absence of various concentrations of the peptoids.

We used GST pull-down assay to determine whether ACE2P1 and ACE2P2 can block the interaction between SARS-CoV-2 spike protein and ACE2. When GST-tagged SARS-CoV-2 spike protein binds to ACE2, the protein complex is captured on beads with immobilized glutathione and pulled down from the solution. We then detected ACE2 in the complex by Western blot. As shown in Figure 2a–c, ACE2P1D1 of ACE2P2D1 dimers prevented the binding of SARS-CoV-2 spike protein to ACE2. Effective blocking of spike-ACE2 interaction was achieved at doses as low as 1 µM of these dimers. In contrast, both ACE2P1 and ACE2P2 monomers increased the binding of spike protein to ACE2, an interesting opposite effect. The D614G mutation in the spike protein makes the virus more contagious [48]. We also found that both ACE2P1D1 of ACE2P2D1 blocked the interaction of D614G spike protein and ACE2, although the effect seems lesser than on the unmutated version of the spike protein (Figure 2d). Of the two peptoids, ACE2P1D1 seems more effective than ACE2P2D1 in blocking the interaction of D614G spike protein and ACE2.

### 2.4. Blocking SARS-CoV-2 Pseudotyped Virus Entry into Human Cells

**Figure 3:**
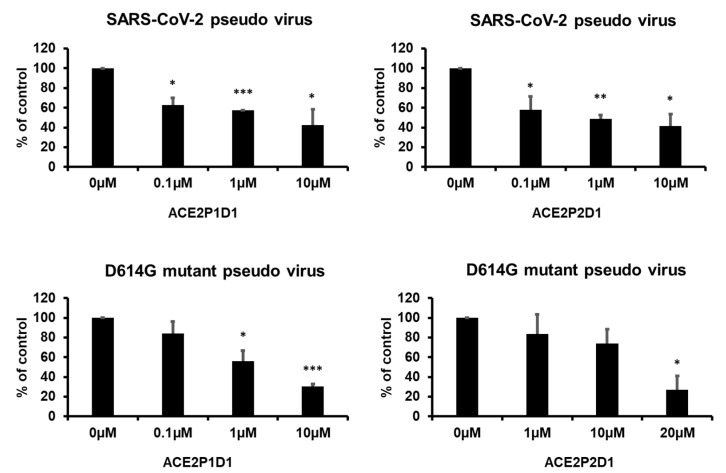
ACE2P1 and ACE2P2 dimers prevented SARS-CoV-2 pseudovirus infection of human cells. (**a**,**b**) NCI-H1299 cells were pretreated with PBS or various concentrations of ACE2P1D1 or ACE2P2D1 for 1 h and then infected with pseudovirus for 24 h. Infection was detected with luciferase reporter assay at 72 h. (**c**,**d**) NCI-H1299 cells were pretreated with PBS or various concentrations of ACE2P1D1 or ACE2P2D1 for 1 h and then infected with D614G mutant pseudovirus for 24 h. Infection was detected with luciferase reporter assay at 72 h. Each bar represents the mean + standard deviation (*n* = 2/group). * *p* < 0.05, ** *p* < 0.01, and *** *p* < 0.001.

To test the capability of ACE2P1D1 and ACE2P2D1 in blocking SARS-CoV-2 infection, we used a pseudotyped virus to conduct assays in a regular BSL-2 lab. This is a lentiviral vector-based pseudovirus that contains the luciferase gene for convenient measurement of the virus infectivity. The pseudotyping was performed by co-transfecting two lentiviral plasmids (carrying the lentiviral backbone and the rep-cap gene, respectively) with another plasmid that contains the full-length spike gene of SARS-CoV-2. After the titer was determined, the generated pseudovirus was used to infect human cells in the presence or absence of the peptoids. As shown in Figure 3a,b, ACE2P1D1 and ACE2P2D1 were effective in inhibiting SARS-CoV-2 pseudovirus infection in human lung cancer NCI-H1299 cells (which express ACE2). Furthermore, ACE2P1D1 and ACE2P2D1 effectively blocked the infection of SARS-CoV-2 pseudovirus containing the D614G mutant spike protein (Figure 3c,d). ACE2P1D1 was more effective than ACE2P2D1 in blocking the SARS-CoV-2 pseudovirus containing the D614G mutant spike protein.

### 2.5. ACE2P1D1 and ACE2P2D1 Do Not Inhibit the Enzymatic Activity of ACE2

**Figure 4:**
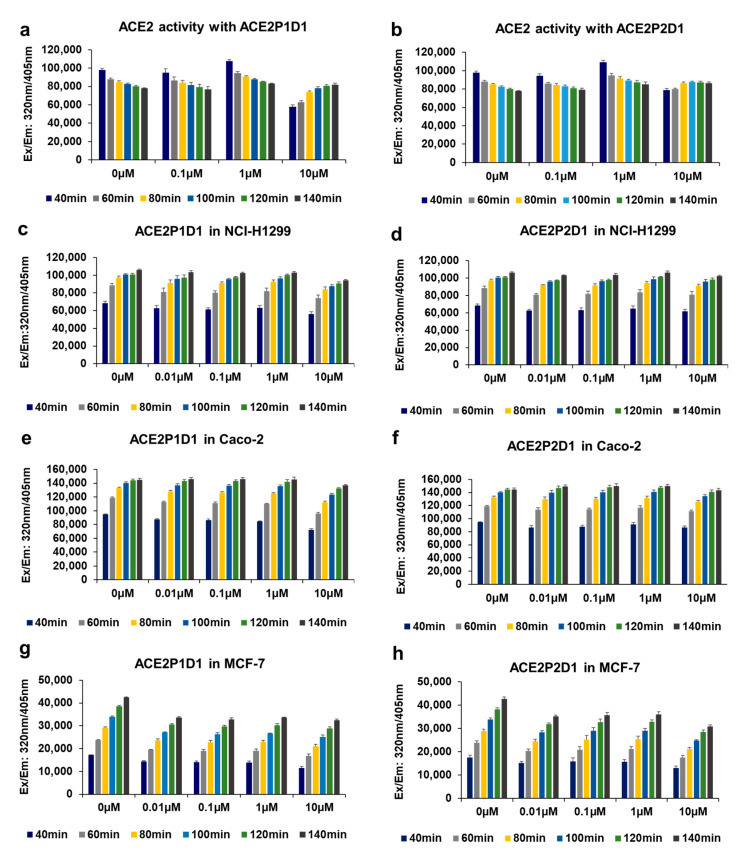
ACE2P1D1 and ACE2P2D1 do not affect the enzymatic activity of ACE2. (**a**,**b**) Assays were run at 37 °C in ACE2 reaction buffer containing 10 μM Mca-YVADAPK(Dnp) with 0.1 µg of ACE2 protein and various concentrations of ACE2P1D1 and ACE2P2D1. Fluorescence (excitation 320 nm and emission 405 nm) was measured in a microplate reader. Cleavage of the substrate by ACE2 enzyme produces fluorescence, which indicates ACE2 enzymatic activity. (**c**–**h**) Human cell lines NCI-H1299, Caco-2, and MCF-7 were treated with various concentrations of ACE2P1D1 or ACE2P2D1, in the presence of cOmplete™ Protease Inhibitor Cocktail (40 µL/mL) and 10 μM captopril. ACE2 activity was measured as described in (**a**,**b**), using 40 µg of cell lysate per each cell line. Each bar represents the mean + standard deviation (*n* = 3/group).

ACE2 is an enzyme which lowers blood pressure by catalyzing the hydrolysis of angiotensin II [49]. Since its enzyme activity is beneficial to blood pressure regulation, it is important that our compounds do not inhibit ACE2 enzymatic activity. To determine if ACE2P1D1 and ACE2P2D1 interfere with the normal functions of ACE2, we examined the effects of the peptoids on ACE2 enzyme activity. ACE2 enzyme activity was measured by fluorescence generated from the cleavage of the substrate Mca-YVADAPK(Dnp) by ACE2. As shown in Figure 4a,b, ACE2P1D1 and ACE2P2D1 did not affect ACE2 enzyme activity when they were incubated with recombinant ACE2 protein. We then examined the effects of ACE2P1D1 and ACE2P2D1 on ACE2 activity in cultured cells. For measuring ACE2 activity in cells, cell homogenates were used. To prevent hydrolysis of the substrate by a range of nonmetalloprotease enzymes from the cells, cOmplete™ Protease Inhibitor Cocktail (Roche/Sigma—40 µL/mL) was added to the cell homogenates. To eliminate the effects of ACE on the substrate, ACE inhibitor captopril (10 µM) was added to the assay. As shown in Figure 4c–h, ACE2P1D1 and ACE2P2D1 had no effects on ACE2 activity in cultured NCI-H1299, Caco-2, and MCF-7 cells.

### 2.6. ACE2P1D1 and ACE2P2D1 Do Not Decrease ACE2 Expression

**Figure 5:**
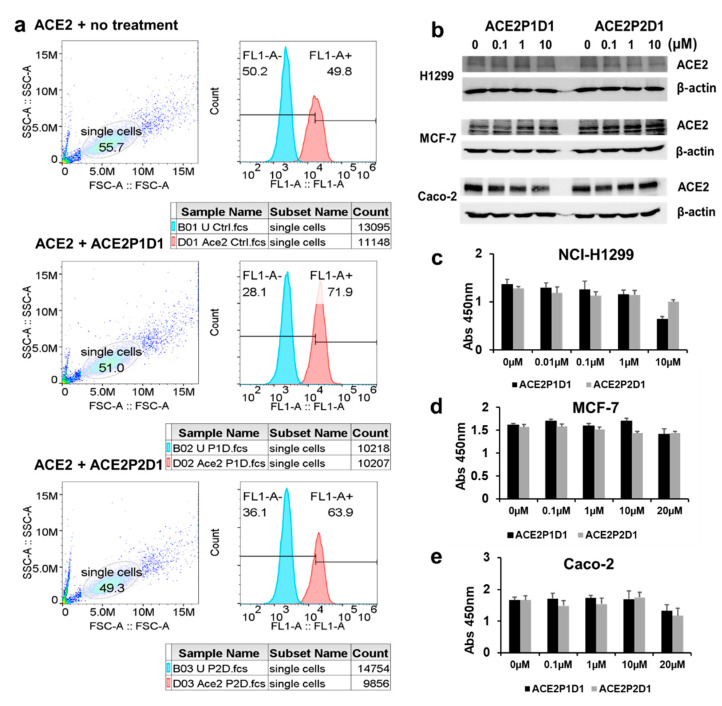
ACE2P1D1 and ACE2P2D1 do not decrease ACE2 expression and are not toxic to cells. (**a**) NCI-H1299 cells were treated with PBS or 10 µM of ACE2P1D1 or ACE2P2D1 for 48 h. ACE2 expression was detected by flow cytometry. ACE2 expression was not changed in ACE2P1D1 or ACE2P2D1 treated cells. (**b**) NCI-H1299, MCF-7, and Caco-2 cells were treated with various concentrations of ACE2P1D1 or ACE2P2D1 for 48 h. Total ACE2 levels in cells were analyzed by Western blot. The treatment of ACE2P1D1 or ACE2P2D1 had no effect on ACE2 protein levels. For each cell line, blots were cropped from different parts of the same gel. (**c**–**e**) NCI-H1299, MCF-7, and Caco-2 cells were treated with various concentrations of ACE2P1D1 or ACE2P2D1 for 48 h. Cell viability was measured by WST-1 assay. The treatment of ACE2P1D1 or ACE2P2D1 had no effect on the cell viability of NCI-H1299, MCF-7, and Caco-2 cells within the peptoid concentrations tested up to 20 µM. Each bar represents the mean + standard deviation (*n* = 3–5/group).

Binding of ligands to a cell surface receptor may decrease the expression level of the receptor through processes such as endocytosis. We assessed the effects of ACE2P1D1 and ACE2P2D1 on ACE2 expression on the cell surface. Control and peptoid-treated NCI-H1299 cells were incubated on ice with goat anti-ACE2 antibody (R&D Systems) and then incubated with FITC-labeled anti-goat IgG antibody. Labeled cells were analyzed by flow cytometry. As shown in Figure 5a, treatment with ACE2P1D1 and ACE2P2D1 did not decrease cell surface expression of ACE2 in H1299 cells. Similarly, treatment with ACE2P1D1 and ACE2P2D1 did not decrease the cell surface expression of ACE2 in Caco-2 and MCF-7 cells (Supplementary Figures S12 and S13). Next, we assessed the effects of ACE2P1D1 and ACE2P2D1 on total cellular levels of ACE2 protein. As shown in Figure 5b, total ACE2 protein levels did not change after ACE2P1D1 and ACE2P2D1 treatments in NCI-H1299, Caco-2, and MCF-7 cells.

### 2.7. ACE2P1D1 and ACE2P2D1 Are Not Toxic to Human Cells

We assessed whether ACE2P1D1 and ACE2P2D1 are toxic to the cells. We seeded and treated increasing concentrations of ACE2P1D1 and ACE2P2D1 on NCI-H1299, Caco-2, and MCF-7 cells. The standard WST-1 assay was used to measure the cell viability. As shown in Figure 5c–e, treatment with both compounds had no effects on cell viability in NCI-H1299, MCF-7, and Caco-2 cells.

## 3. Discussion

Binding of SARS-CoV-2 spike protein S to human ACE2 receptor is the key entry point for SARS-CoV-2 virus infection. Therefore, blocking this interphase of virus spike protein S and ACE2 is an attractive strategy to combat COVID19 pandemic. This has been successfully tested by developing neutralizing antibodies [50] and an aptamer [51] that can occupy the receptor-binding domains (RBDs) of the SARS-CoV-2 spike protein, which effectively blocks ACE2 recognition. However, the rapid development of various mutations in the virus can reduce the effectiveness of these antibodies or any other molecular classes that may be developed to bind to virus RBDs. The other side of this critical interphase is the ACE2 receptor. However, concerns over interfering with the normal physiological functions of ACE2 hinder the interests of directly targeting ACE2 for COVID-19 prevention and treatment. Since the 2003 SARS epidemic, researchers have identified numerous ACE2 binding ligands such as small molecules, peptides, and antibodies that block SARS-CoV virus entry [21]. Peptides derived from the sequence of SARS-CoV spike protein or ACE2 were able to block SARS-CoV spike-ACE2 interaction. These peptides showed antiviral activity against SARS-CoV infection in vitro [14,15,16,17]. However, their effects on ACE2 activity have not been reported and they have not been tested against SARS-CoV-2 virus, whose spike protein is different from SARS-CoV virus (the RBD of SARS-CoV-2 is only about 74% homologous to that of SARS-CoV) [52]. Peptides are also unstable in serum, making them less optimal as drug candidates. Small molecules were also identified to bind to ACE2. The strengths of using small molecules to target ACE2 include better pharmacokinetics and drug-like properties. However, they inhibit the function of ACE2 and their effects in blocking viral infection have not been tested [12,13]. Several monoclonal antibodies targeting the SARS-CoV-2 spike protein have been reported [53,54,55,56] and several are in clinical trials. These antibodies effectively blocked viral infection in animal models. A potential limitation of monoclonal antibodies is the unknown bioavailability of passively infused IgG in tissues affected by the disease, especially the lung, which is a key target of SARS-CoV-2 infection. The antibodies are large (~150 kD in size) molecules. In contrast, our peptoid compounds are 1–4 kD in size. An ACE2 specific antibody was used as a method to show the function of ACE2 as the receptor for the virus [6]. However, the antibody was not tested as therapy to block virus entry. Certain ACE2 targeted antibodies inhibit ACE2 enzymatic activity [57], making them unsuitable for COVID-19 prevention.

In this study, we have identified novel ACE2-binding peptoids that block spike-ACE2 interaction and prevent SARS-CoV-2 pseudotyped virus infection without affecting ACE2 expression and its enzymatic activity. These results suggest that it is possible to safely target ACE2 to prevent and treat COVID-19 and other coronavirus infections. Our ACE2 enzyme activity data, shown in Figure 4, rule out the possibility of our peptoids binding to the ACE2 active site, as our peptoids have no effect on ACE2 activity. There can be two possibilities for potential binding sites for our peptoids on the ACE2 surface. Our peptoids may directly bind to the virus spike protein binding site on ACE2 and may directly block the virus spike protein binding to ACE2. Conversely, our peptoids may recognize a different binding site on ACE2 that has not been validated for any important activity yet but have an antagonizing effect on subsequent conformational changes needed for virus spike protein-ACE2 complex to facilitate virus entry into the cell. Furthermore, the possibility of ACE2 receptor destabilization, degradation, or internalization can be ruled out based on our data shown in Figure 5a,b, which indicates no decrease of ACE2 protein upon peptoid binding.

Our OBTC assay uniquely allows exposing ACE2 receptor to the peptoid library while ACE2 is in its fully natural condition on the cell surface. We believe that these peptoids that were displayed on the library resin bead surface “saw” the ACE2 receptor exactly as it was “seen” by virus spike protein during the evolution to pick its target to enter human cells. The viral spike protein might have chosen the most exposed “tip” of the ACE2 receptor protein to establish its first direct contact [28,29], and the same might have happened with our peptoids displayed on resin beads. This hypothesis suggests that there is a high chance that our peptoids may bind to the spike protein binding site of the ACE2 and physically block the virus-ACE2 interaction. Our data is highly significant as a recent study has shown that the binding of SARS-CoV-2 spike protein caused an increase in ACE2 enzymatic activity [23,58], which may be relevant to the cardiovascular symptoms associated with COVID-19. This finding indicates that ligands binding to virus spike protein binding site on the ACE2 surface can affect the ACE2 active site, despite that these two sites are spatially separated. However, our peptoids have no effects on ACE2 activity (Figure 4) and ACE2 protein expression (Figure 5a,b), and have no toxicity to the three human cell lines that we have tested (Figure 5c–e).

As described in the introduction, peptoids are highly biologically amenable and are easier and economical to optimize. This allows rapid development of formulations and products such as nasal sprays or eye drops using our peptoids, as the main sites of ACE2 receptor exposure are in the nose and the eye. In addition, these peptoids can be developed into conventional drugs (considering the high serum stability and tissue permeability of peptoids) to block the virus spreading inside the body tissues. Therefore, our peptoid lead compounds represent a new strategy to develop a first-line physical defense system to block virus entry using “molecular masks” that bind to the ACE2-virus spike protein interface while having no effect on the ACE2 enzyme active site. An advantage of this approach is that there will be no concern for any viral escape from an ACE2 binding peptoid, which cannot be achieved by the neutralizing approaches against the virus spike protein.

## 4. Materials and Methods

### 4.1. Chemicals and Reagents

TentaGel MB NH₂ resin (particle size: 140–170 µm, loading capacity: 0.2–0.3 mmol/g, 520,000 beads/g) was purchased from Rapp Polymere GmbH (Tuebingen, Germany). Rink amide resin (particle size: 100–200 mesh, loading capacity: 0.3–0.6 mmol/g) was purchased from Chem-Impex International, Inc. (Wood Dale, IL, USA). All Fmoc-protected amino acids and 2-(1H-Benzotriazole-1-yl)-1,1,3,3-tetramethyluronium hexafluorophosphate (HBTU), Hydroxybenzotriazole (HOBt), all primary amines, bromoacetic acid, N,N-diisopropylcarbodiimide (DIC), N,N-diisopropylethylamine (DIPEA), piperidine, trifluoroacetic acid (TFA), cyanogen bromide (CNBr), Triisopropylsialine (TIS), α-cyano-4-hydroxycinnamic acid, acetonitrile (ACN), hydrochloric acid (HCl), dichloromethane (DCM), and N,N-dimethylformamide (DMF), were obtained from MilliporeSigma (Burlington, MA, USA). GIBCO enzyme free cell dissociation buffer and Qtracker Cell Labeling Kits were obtained from ThermoFisher Scientific (Waltham, MA, USA). All chemical reagents and solvents from commercial sources were used without further purification. Five-ml disposable reaction columns (CEM Corporation, Matthews, NC, USA) were used as reaction vessels for solid-phase synthesis. Syntheses of peptoids under microwave conditions were performed in a 1000 W microwave oven with 10% power. All purifications were completed on a Waters HPLC system (Waters Corporation, Milford, MA, USA). Mass spectra were recorded on an Applied Biosystems Voyager DE Pro mass spectrometer using α-cyano-4-hydroxycinnamic acid as the matrix.

### 4.2. Library Synthesis

The basic structure of the library consists of two amino acids followed by 6-mer diversified peptoid region. TentaGel MB NH2 6 g (140–170 μm; substitution: 0.2–0.3 mmol/g resin; Rapp Polymere, Tübingen, Germany) were swelled in N,N-dimethylformamide (DMF) for 30 min at room temperature in a 5 mL reaction column (Intavis AG, Tübingen, Germany) (200 mg of resin in each column). The DMF was drained from the reaction vessels, the resin was first coupled to Fmoc-Met-OH (5.0 equivalent (equiv.)) using 5.0 equiv. 2-(1H-Benzotriazole-1-yl)-1,1,3,3-tetramethyluronium hexafluorophosphate (HBTU) and 5.0 equiv. Hydroxybenzotriazole (HOBt) as coupling reagents in the presence of 10.0 equiv. of N,N-diisopropylethylamine (DIPEA) for overnight shaking. Fmoc group was removed by treating the resins with 20% piperidine in DMF twice for 10 min. After washing the resins, Fmoc-Lys (Boc)-OH was added (for 2.0 h reaction time) and Fmoc group was removed as described previously. The rest of the synthesis was achieved using the split-pool synthesis protocol. A total of 10 different amines were chosen for the library, including N-Boc-1,4-butanediamine, allylamine, isobutylamine, 2-methoxyethylamine, 3-isopropoxypropylamine, β-alanine, (R)-(+)-α-methylbenzylamine, 4-methoxybenzylamine, piperonylamine, and furfurylamine. The resins were equally distributed into 10 batches for microwave-assisted peptoid synthesis steps. Each of the reaction batches was treated with 1.0 M bromoacetic acid in anhydrous DMF (1.0 mL) and 1.5 M DIC in anhydrous DMF (1.0 mL), gently shaken for 30 s, and microwaved (1000 W) for 15 s with the power set at 10%. The beads were shaken again for 30 s and microwaved another round as described above. The reaction columns were drained and washed with DMF (2.0 mL × 10 times). Then, each of the reaction batches was treated with 1.0 mL of 2.0 M solution of one primary amine (10 different batches treated with 10 different amines) and was placed on a shaker for 2.0 h at 25 °C. The resins were washed, pooled, and divided again equally into 10 batches and subjected to an addition of the next peptoid residue. This procedure was repeated until 6-mer peptoid region was completed. At the end of synthesis, the beads were washed with dichloromethane (DCM) (2.0 mL × 3 times) and treated with 2.5 mL of 95% trifluoroacetic acid (TFA), 2.5% water, and 2.5% Triisopropylsialine (TIS) on the shaker for 2 h to remove the side chain protection and were neutralized with 10% diisopropylethylamine in DMF. The reaction vessel was drained, washed with DMF (2.0 mL × 3 times), and stored in anhydrous DMF at 4 °C.

### 4.3. On Bead Two Color Binding Assay for Combinatorial Library Screen

A total of 50,000 peptoid library beads were washed two times in DMEM medium containing 10% FBS (media) and then incubated in 1.0 mL (DMEM + 10% FBS) for 1.0 h in a polypropylene tube. ACE2 positive MCF-7cells and ACE2 negative MCF-7 cells were removed from culture plates with GIBCO enzyme-free cell dissociation buffer (ThermoFisher Scientific, Cat # 13151014) at 2.0 mL per plate for 20 min at 37 °C. Cells were washed and suspended in DMEM + 10% FBS media. Cells were counted and distributed in 1.5 mL microcentrifuge tubes with 1.0 × 106 cells in 1.0 mL of media. Then, the cell labeling procedure was conducted as follows: 1.0 μL each of Qtracker reagent A and B were mixed in 1.5 mL microcentrifuge tubes and incubated for 5.0 min at room temperature. Media (0.2 mL) was added to each tube and vortexed for 30 s. A measured amount of 1.0 × 106 cells were added to each tube containing the labeling solution and incubated at 37 °C for 60 min. ACE2 positive MCF-7 cells were labeled with Qtracker 655 (red color) (ThermoFisher Scientific, Cat# Q25021MP, Waltham, MA, USA) and ACE2 negative MCF-7 cells labeled with Qtracker 565 (green color) (ThermoFisher Scientific, Cat# Q25031MP, Waltham, MA, USA). Cells were washed twice and suspended in 1.0 mL of DMEM + 10% FBS media. Labeled cells were visualized with long-pass filter of the BX-53F fluorescence microscope (Olympus, Waltham, MA, USA) with a color camera. Both cell types were mixed thoroughly and pipetted up and down several times to break the clumps. A cell suspension mixture of 2.0 mL was added to the tube containing 50,000 beads and incubated at room temperature with gentle shaking for 1.0 h. During incubation, cell binding to the beads were checked periodically at about 15 min intervals to ensure not to over equilibrate, which could increase non-specific binding of cells to the beads. The beads were gently washed two times with DMEM + 10% FBS media and visualized under the fluorescent microscope using the long-pass filter.

### 4.4. Isolation and Preparation of Beads for Sequencing

Single bead containing fluorescently tagged red cells was identified using a fluorescent microscope under 10× objective magnification and removed manually with a 20 μL pipette with medium size pipette tips. Selected beads were washed three times with 1.0% SDS and boiled in the same solution for 10 min to strip off bound cells and proteins. Finally, the beads were washed three times with water. To cleave the compound from the bead and prepare it for MS/MS sequencing, cleaving solution was prepared; thus, 30 μL of cyanogen bromide (CNBr) (5.0 M in Acetonitrile (ACN)) was added to 1.0 mL of 0.1 N HCl. Cleaving solution (50 μL) was added to the 1.5 mL tube, which contained the single isolated bead. The tube was incubated at 25 °C for 4.0 h. The solution was evaporated using a freeze dryer (SP Scientific, Gardiner, NY, USA), and the cleaved compound was suspended in 20 μL of water. MS/MS sequencing data was obtained using AB Sciex TOF/TOF 5800 machine.

### 4.5. Validation of on Bead Two Color Binding Screening Results

After identifying the compound (ACE2P1 and ACE2P2) with MS/MS sequencing, they were resynthesized on TentaGel MB NH₂ beads. Three tubes of 25,000 beads with each compound (containing ACE2P1 and ACE2P2 compounds) were prepared by washing and incubating for 1.0 h in DMEM + 10% FBS. Two million cells each of ACE2 positive MCF-7 cells were stained red in color using Qtracker 655, and ACE2 negative MCF-7 cells were stained in green color using Qtracker 565. One million ACE2 positive MCF-7 cells (red cells) were suspended in 1.0 mL of DMEM + 10% FBS media and were added to another 25,000 beads-containing tube. One million ACE2 negative MCF-7 cells (green cells) were suspended in 1.0 mL of DMEM + 10% FBS media and were added to 25,000 beads-containing tube. To create a mixture of cells, 0.5 × 106 of red cells and 0.5 × 106 green cells were mixed together and suspended in 1.0 mL of DMEM + 10% FBS media and were added to the third tube containing 25,000 beads. The cells were incubated with the beads for 1.0 h at room temperature. The beads were gently washed twice with DMEM + 10% FBS media and visualized under the fluorescent microscope using the long-pass filter.

### 4.6. Synthesis of ACE2P1

ACE2P1 was synthesized on Rink amide resin (particle size: 100–200 mesh, loading capacity: 0.3–0.6 mmol/g)/TentaGel MB NH₂ resin. An amount of 100 mg of resin was taken in 5 mL reaction column, the resin was swelled in dimethylformamide (DMF) for 1.0 h prior to use, and the Fmoc group was deprotected by treating the resin with 2.0 mL of 20% piperidine solution in DMF twice for 10 min each. The resin was first coupled to Fmoc-Met-OH (5 equiv.) using 5.0 equiv. HBTU and 5.0 equiv. HOBt as coupling reagents in the presence of 10.0 equiv. of DIPEA overnight. Fmoc was removed with the method described above. Subsequent amino acid Fmoc-Lys(Boc)-OH was introduced using the same peptide-coupling protocol (HBTU/HOBt/DIPEA), washing 10 times with DMF between each reaction. After removing the Fmoc group as described above, six peptoid residues were then coupled using a two-step peptoid coupling procedure (acylation and amination) under a microwave-assisted synthesis protocol. For the acylation step, beads were treated with 1.0 M bromoacetic acid (1.0 mL) and 1.5 M DIC (1.0 mL) and microwaved at 10% power (2 × 15 s) with gentle shaking in between for 30 s. After washing with DMF, beads were treated with 1.0 mL of (R)-(+)-α-methylbenzylamine (2.0 M), and coupling was performed by shaking at 25 °C for 2 h. The procedure was repeated to attach the remaining five residues: allylamine, 4-methoxybenzylamine, (R)-(+)-α-methylbenzylamine, 2-methoxyethylamine, and isobutylamine, in order. At the end, beads were washed with dichloromethane (DCM) and dried under vacuum before cleavage. Beads were then treated with a cleaving cocktail of TFA/H2O/TIS (95%/2.5%/2.5%) for 2.0 h. The crude compound was then purified using HPLC (Waters Corporation, Milford, MA, USA) and analyzed by MALDI-TOF (Applied Biosystems Voyager DE Pro mass spectrometer, Waltham, MA, USA). Structures are shown in Supplementary Materials (Figures S4 and S5).

### 4.7. Synthesis of ACE2P1D1

ACE2P1D1 was synthesized using a protocol similar to ACE2P1. An amount of 100 mg of Rink amide resin was taken in 5 mL reaction column, the resin was swelled in dimethylformamide (DMF) for 1.0 h prior to use, and the Fmoc group was deprotected; the resin was first coupled to Fmoc-Lys(Fmoc)-OH (5 equiv.) using 5.0 equiv. HBTU and 5.0 equiv. HOBt as coupling reagents in the presence of 10.0 equiv. of DIPEA overnight. Next, both Fmoc groups were deprotected simultaneously, the Fmoc deprotection of both amine groups produced two NH2 function groups to build two copies of ACE2P1 simultaneously to obtain a homo-dimer. The resin was coupled with Fmoc-Met-OH and Fmoc-Lys(Boc)-OH using the peptide-coupling protocol (HBTU/HOBt/DIPEA). After removing the Fmoc, six peptoid residues were then coupled using a two-step peptoid coupling procedure (acylation and amination) under a microwave assisted synthesis protocol. The sequence of peptoid residues was: (R)-(+)-α-methylbenzylamine, allylamine, 4-methoxybenzylamine, (R)-(+)-α-methylbenzylamine, 2-methoxyethylamine, and isobutylamine, in order). Structure is shown in Supplementary Materials (Figure S6).

### 4.8. Synthesis of Biotin-ACE2P1D1

Synthesis of Biotin-ACE2P1D1 was performed using the similar protocol described for ACE2P1. The sequence for amino acid residues for Biotin-ACE2P1D1 were Fmoc-Cys(Trt)-OH, Fmoc-Lys(Fmoc)-OH, Fmoc-Met-OH, and Fmoc-Lys(Boc)-OH. After removing the Fmoc, six peptoid residues were then coupled using a two-step peptoid coupling procedure (acylation and amination) under a microwave-assisted synthesis protocol. The sequence of peptoid residues was: (R)-(+)-α-methylbenzylamine, allylamine, 4-methoxybenzylamine, (R)-(+)-α-methylbenzylamine, 2-methoxyethylamine, and isobutylamine, in order. The compound was cleaved from the beads by treating with TFA/H2O/TIS (95%/2.5%/2.5%) for 2.0 h. Cysteine-attached ACE2P1D1 was obtained by purifying the mixture using HPLC. Biotin-maleimide [N-Biotinoyl-N′-(6-maleimidohexanoyl)hydrazide] was added to the purified portion of Cysteine attached ACE2P1D1 in 1:1 equivalent ratio in water and the pH the solution was adjusted to 7, the mixture was allowed to stir overnight at 4 °C and the compound was purified using HPLC to obtain Biotin-ACE2P1D1. Structure is shown in Supplementary Materials (Figure S7).

### 4.9. Synthesis of ACE2P2

ACE2P2 was synthesized using peptide-coupling and peptoid coupling protocol similar to ACE2P1. The sequence for amino acids residues for ACE2P2D1 were Fmoc-Met-OH followed by Fmoc-Lys(Boc)-OH. Next, six peptoid residues were then coupled using a two-step peptoid coupling procedure. The sequence of peptoid residues was: 2-methoxyethylamine, isobutylamine, isobutylamine, 3-isopropoxypropylamine, 4-methoxybenzylamine and 4-methoxybenzylamine, in order. Structures are shown in Supplementary Materials (Figures S8 and S9).

### 4.10. Synthesis of ACE2P2D1

ACE2P2D1 was synthesized using peptide-coupling and peptoid coupling protocol similar to ACE2P1. The sequence for amino acids residues for ACE2P2D1 were Fmoc-Lys(Fmoc)-OH followed by Fmoc-Met-OH and Fmoc-Lys(Boc)-OH, in order. Next, six peptoid residues were then coupled using a two-step peptoid coupling protocol. The sequence of peptoid residues was: 2-methoxyethylamine, isobutylamine, isobutylamine, 3-isopropoxypropylamine, 4-methoxybenzylamine and 4-methoxybenzylamine, in order. Structure is shown in Supplementary Materials (Figure S10).

### 4.11. Synthesis of Biotin-ACE2P2D1

Synthesis of Biotin-ACE2P2D1 was performed using the similar protocol described for ACE2P1. The sequence for amino acids residues for Biotin-ACE2P2D1 were Fmoc-Cys(Trt)-OH, Fmoc-Lys(Fmoc)-OH, Fmoc-Met-OH and Fmoc-Lys(Boc)-OH. After removing the Fmoc, six peptoid residues were then coupled using a two-step peptoid coupling procedure (acylation and amination) under a microwave-assisted synthesis protocol. The sequence of peptoid residues was: 2-methoxyethylamine, isobutylamine, isobutylamine, 3-isopropoxypropylamine, 4-methoxybenzylamine and 4-methoxybenzylamine, in order. The compound was cleaved from the beads by treating with TFA/H2O/TIS (95%/2.5%/2.5%) for 2.0 h. Cysteine attached ACE2P2D1 was obtained by purifying the mixture using HPLC. Next, Biotin-maleimide [N-Biotinoyl-N′-(6-maleimidohexanoyl)hydrazide] was added as described for Biotin-ACE2P1D1 to obtain Biotin-ACE2P2D1. Structure is shown in Supplementary Materials (Figure S11).

### 4.12. ELISA-Like Assay

An amount of 100 µL/well of recombinant human angiotensin-converting enzyme 2 (ACE2) (His-tag) (RayBiotech, Cat# 230-30165-100, Peachtree Corners, GA, USA) protein (1 µg/mL in TBS) was added to nickel coated white 96-well plates (ThermoFisher Scientific, Cat# 15242, Waltham, MA, USA) and incubated for 1.0 h (hr) at room temperature (RT) with slow shaking. Wells were washed 3 × 200 µL of TBST, and 200 µL of blocking buffer (2.0% BSA in TBS) was added and the plate was incubated for 1.0 hrs at RT. Biotinylated ACE2P1D1/Biotinylated ACE2P2D1 was added with varying concentrations (diluted in blocking buffer) (100 µL/well) and incubated for 1.0 h with slow shaking, and then washed 3 × 200 µL with TBST and added 100 µL of Streptavidin-HRP (1:1000) (dilution prepared in blocking buffer) (BioLegend, Cat# 405210, San Diego, CA, USA) and incubated for 1.0 h at RT with slow shaking. Washed the wells with TBST 6 times (200 µL each) and 100 µL of SuperSignal ELISA Pico Chemiluminescent Substrate (1:1 mixture) (Thermo Fisher Scientific, Cat# 37070, Waltham, MA, USA) was added to each well and luminescence signal was detected at all wavelengths.

### 4.13. Cells and siRNA Transfection

The cell lines MCF-7, H1299, and Caco-2 were purchased from American Type Culture Collection. Cells were maintained in Dulbecco’s Modified Eagle’s Medium (Corning, Cat# 10-017-CV, Tewksbury, MA, USA), supplemented with 100 units/mL penicillin, 100 µg/mL streptomycin, and 10% fetal bovine serum (Corning, Cat# 35-011-CV, Tewksbury, MA, USA) at 37 °C with 5% CO_2_. For siRNA transfection, MCF-7 cells were seeded (2.5 × 106/dish) in a 10cm cell culture dish one day before transfection and treated with human ACE2 siRNA (Dharmacon, Cat # L-005755-00-0005, Lafayette, CO, USA) for 24 h with X-tremeGENE siRNA transfection reagent (Roche, Ref# 04476093001, St. Louis, MO, USA) according to the manufacturer’s instructions.

### 4.14. Western Blot Analysis

Cells were lysed in RIPA buffer (1% NP-40, 0.5% sodium deoxycholate, 0.1% SDS in PBS). Complete protease inhibitor cocktail (Roche, St. Louis, MO, USA) was added to lysis buffer before use. Protein concentration was determined by Bio-Rad DC protein assay (Bio-Rad, Hercules, CA, USA). Protein samples were subjected to SDS-PAGE and transferred to nitrocellulose membrane. The membrane was blocked in 5% non-fat milk in PBST overnight and incubated with primary antibody and subsequently with appropriate horse radish peroxidase-conjugated secondary antibody. Signals of targeted proteins were detected by the Immun-Star HRP peroxide Luminol/Enhancer (Bio-Rad, Hercules, CA, USA) and recorded on ChemiDoc Touch Imaging System (Bio-Rad, Hercules, CA, USA). Anti-hACE2 (human ACE2 receptor) antibody was purchased from R&D system, Inc. (Cat# AF933, Minneapolis, MN, USA). Full western blot data are given in Supplementary Material (Figures S14–S16).

### 4.15. In Vitro Pull-Down Assay

10 nM of recombinant human ACE2 protein (R&D system, Cat# 933-ZN-010, Minneapolis, MN, USA) was incubated with ACE2P1 and ACE2P2 conjugated beads in RIPA buffer at 4 °C for 2 hrs. The beads were then washed with RIPA buffer for 3 times, and the binding proteins were eluted with 1% SDS at 95 °C for 5 min. The yielded lysates were then mixed with 4× Laemmli Sample Buffer (loading buffer) (Bio-Rad, Cat# 1610747, Hercules, CA, USA). The samples were then applied onto 8% SDS-PAGE gel. The gels were consequently subjected to Western blotting analysis.

### 4.16. GST Protein Interaction Pull-Down Assay

Pierce GST Protein Interaction Pull-down kit (Thermo Fisher Scientific, Cat# 21516, Waltham, MA, USA) was used to perform GST pull-down assay following manufacturer’s protocol. Columns containing glutathione agarose resin were washed 5 times by centrifugation at 1250× *g* for 1 min. A total of 0.1 µg of GST-tagged recombinant SARS-CoV-2 spike protein (Proteintech, Cat# Ag30689, Rosemont, IL, USA) was added to the column and incubated at 4 °C for 1 h. While incubating the spike protein, 0.1 µg of recombinant ACE2 protein (R&D system, Cat# 933-ZN-010, Minneapolis, MN, USA) was incubated with each of ACE2P1, ACE2P2, ACE2P1D1, and ACE2P2D1 in different concentrations (0.1 µM, 1 µM, and 10 µM) in 400 µL washing solution at cold room for 1 h by tube rotator with a gentle motion. After incubation, the columns with recombinant spike protein were washed 3 times by centrifugation at 1250× *g* for 1 min. ACE2 protein alone or ACE2 preincubated with ACE2P1, ACE2P2, ACE2P1D1, and ACE2P2D1 were added into each column, and incubated at 4 °C for 2 h (total volume of 400 µL for each sample). After incubation, they were washed with 400 µL of washing solution for a total of 3 washes. An amount of 200 µL of elution buffer (10 mM Glutathione) was added into each column and incubated for 5 min at room temperature. The samples were centrifuged at 1250× *g* for 1 min and placed on ice. SDS-PAGE gel (8%) was prepared, and the eluted samples were mixed with 4× Laemmli Sample Buffer (loading buffer) (Bio-Rad, Cat# 1610747, Hercules, CA, USA). Prepared samples were applied onto the gel. Western blot was performed using anti-hACE2 antibody from R&D system (Cat# AF933, Minneapolis, MN, USA).

### 4.17. His-D614G Spike—ACE2 Protein Interaction Pull-Down Assay

Pierce His Protein Interaction Pull-down kit (Thermo Fisher Scientific, Cat# 21277, Waltham, MA, USA) was used to perform His pull-down assay following manufacturer’s protocol. Columns containing HisPur Cobalt resin were washed a total of 5 times by centrifugation at 1250× *g* for 1 min. An amount of 0.1 µg of His-tagged recombinant SARS-CoV-2 S1 (D614G) protein (Sino Biological US Inc., Cat# 40591-V08H3, Wayne, PA, USA) was added to the column and incubated at 4 °C for 1 h. While incubating the spike protein (D614G), 0.1 µg of recombinant ACE2 protein (R&D system Inc., Cat# 933-ZN-010, Minneapolis, MN, USA) was incubated with each ACE2P1D1 and ACE2P2D1 in different concentrations (10 µM, and 100 µM) in 400 µL washing solution at cold room for 1 h by tube rotator with a gentle motion. After incubation, the columns with recombinant spike protein were washed for 3 times by centrifugation at 1250× *g* for 1 min. ACE2 protein alone or ACE2 preincubated with ACE2P1D1 and ACE2P2D1 were added into each column and incubated at 4 °C for 2 h (total volume of 400 µL for each sample). After incubation, they were washed with 400 µL of washing solution for a total of 3 washes. An amount of 200 µL of elution buffer was added into each column and incubated for 5 min at room temperature. The samples were centrifuged at 1250× *g* for 1 min and placed on ice. SDS-PAGE gel (8%) was prepared, and the eluted samples were mixed with 4× Laemmli Sample Buffer (loading buffer) (Bio-Rad, Cat#1610747, Hercules, CA, USA) and heated for 5 min at 95 °C. Prepared samples were applied onto the gel. Western blot was performed using anti-hACE2 antibody from R&D system(Cat# AF933, Minneapolis, MN, USA).

### 4.18. ACE2 Enzyme Activity Assay

ACE2 enzyme activity was determined using recombinant ACE2 protein (R&D system Inc., Cat# 933-ZN-010, Minneapolis, MN, USA) alone or in the presence of ACE2P1D1 and ACE2P2D1 in different concentrations (0.1 µM, 1 µM, and 10 µM), and Mca-Y-V-A-D-A-P-K (Dnp)-OH, Fluorogenic Peptide Substrate VI from R&D System, Inc. (Cat# ES007, Minneapolis, MN, USA). The reaction buffer was prepared using 1M NaCl, 0.5 mM ZnCl_2_, 75 mM Tris, protease inhibitor (40 µL/mL) (Roche, Ref# 11836153001, St. Louis, MO, USA), and 10 µM captopril (Enzo Life Sciences Inc., Cat# ALX-270-212-G001, Farmingdale, NY, USA) with pH 7.5. 0.1 µg of recombinant human ACE2 protein and 10 µM of substrate were used for a total volume of 100 µL. ACE2 protein and each concentration of ACE2P1D1 and ACE2P2D1 were first incubated in 96-well black plate (Corning, Cat# 3915, Tewksbury, MA, USA) on the shaker at room temperature for 20 min in 60 µL volume of each sample. Then, the 10 µM of substrate was added in each sample (40 µL of volume) and incubated at 37 °C for total 140 min. ACE2 enzyme activity was measured every 20 min by fluorescence plate reader (BioTek Cytation 5 cell Imaging Multi-Mode Reader, Winooski, VT, USA) with excitation at 320 nm and emission at 405 nm.

### 4.19. Flow Cytometry Analysis

To evaluate the impact of compounds to ACE2 upon binding in cell lines, MCF-7, NCI-H1299, and Caco-2 cell cultures reaching 70% confluences were incubated with 10 μM ACE2P1D1 and ACE2P2D1 for 48 h and then harvested using trypsin. Briefly, cells were centrifuged and washed with FACS buffer and then fixed by methanol on ice. Cells were suspended with a primary antibody-staining mixture using 0.5 μg (Human/Mouse/Rat/Hamster ACE-2 Antibody, R&D Systems, Inc., Cat# AF933, Minneapolis, MN, USA) per sample. Samples were incubated at room temperature for 1 h followed by washing and secondary antibody (mouse anti-goat IgG-FITC, Santa Cruz, Cat# sc-2356, Dallas, TX, USA) staining. Samples were incubated for another hour and washed before being analyzed using Accuri™ C6 (BD Biosciences, Franklin Lakes, NJ, USA). Data analysis was conducted by FlowJo™ v10 software (v10.5.2, FlowJo. LLC, Ashland, OR, USA).

### 4.20. Protein Thermal Shift Assay

Thermal shift assay was performed using 5 µg of recombinant ACE2 protein from Ray Biotech Life, Inc. (Cat# 230-30165, Peachtree Corners, GA, USA) and 1 µM or 10 µM of ACE2P1D1 or ACE2P2D1 with the Protein Thermal Shift Dye Kit (Thermo Fisher Scientific, Ref# 4461146, Waltham, MA, USA) and QuantStudio 3 Real-Time PCR Systems (Applied Biosystems by Thermo Fisher Scientific, Waltham, MA, USA), following the manufacturer’s protocol. Protein Thermal Shift Software v1.3 (Applied Biosystems by Thermo Fisher Scientific, Waltham, MA, USA) was used for analyzing the data.

### 4.21. Synthesis and Production of SARS-CoV-2 and D614G Mutant Pseudovirus 

SARS-CoV-2 spike sequence (GenBank: MN908947.3) was optimized for human expression and synthesized by GenScript (Piscataway, NJ, USA) with a HA tag at N-terminal. D614G spike coding sequence was modified with a D614G point mutation and a truncation of 19 amino acids at the end of C terminus, cloned into pCDNA 3.1 vector (Invitrogen). To produce D614G spike pseudovirus for reporter assay, the lentivector reporter pSIN-Luc (containing luciferase as the marker gene) and lentiviral vector packaging plasmid psPAX2 (containing HIV gag and pol genes) were co-transfected with D614G spike at a ratio of 4:3:1 by lipofectamine 2000 (Thermo Fisher Scientific, Waltham, MA, USA) in 10 cm dishes of HEK293T cells (ATCC, Manassas, VA, USA). The virus supernatant was harvested at 48 h after transfection and stored at -800C after removing cell debris with a 0.45 µM filter.

### 4.22. Pseudotyped Virus Infection/Blocking Assay

ACE-2 positive H1299 cells were pretreated with PBS or various concentrations of ACE2P1D1 or ACE2P2D1 for 1 h and then infected with a given amount of titrated pseudovirus for 24 h. After removing the medium at 72 h incubation at 37 °C, luciferase assay was performed with Bright-Glo™ Luciferase Assay System (Promega, Madison, WI, USA) and measured with a luciferase reader. Inhibitory effect (%) was calculated by this formula: (treated cell readings/non-treated cell readings) × 100.

### 4.23. Statistical Analyses

Data are expressed as mean ± SEM. Data were analyzed by Student’s t test and were considered statistically significant if *p* < 0.05. The survival rates of the two groups were analyzed using a log-rank test and were considered statistically significant if *p* < 0.05. *p* values are represented as precise p values or generally as * *p* < 0.05, ** *p* < 0.01, and *** *p* < 0.001.

## 5. Patents

There is a patent application resulting from the work reported in this manuscript. Application Number: PCT/US21/26218.

## Supplementary Materials

The following are available online at https://www.mdpi.com/article/10.3390/ijms22168963/s1.
