# Supplementary material for: “Molecular Masks” for ACE2 to Effectively and Safely Block SARS-CoV-2 Virus Entry
Source: Int J Mol Sci. 2021 Aug 20;22(16):8963. doi: 10.3390/ijms22168963 (PMC8396575; doi:10.3390/ijms22168963)
Supplement: Supplementary file 1 [file ijms-22-08963-s001.zip › ijms-1340644-supplementary.pdf]

“Molecular masks” for ACE2 to effectively and safely block SARS-CoV-2 virus entry

Satya Prakash Shukla [1,†], Kwang Bog Cho [1,†], Vineeta Rustagi [1], Xiang Gao [1], Xinping Fu [2], Shaun Xiaoliu Zhang [2], Bin Guo [1,*] and D. Gomika Udugamasooriya [1,3,*]

[1] Department of Pharmacological & Pharmaceutical Sciences; University of Houston, 4849 Calhoun Rd, Houston, TX 77204-5037, USA; spshukla@central.uh.edu (S.P.S.); kcho7@central.uh.edu (K.B.C); vrustagi@uh.edu (V.R.); xgao23@central.uh.edu (X.G.)
[2] Department of Biology and Biochemistry, University of Houston, 3455 Cullen Blvd, Houston, TX 77204-5037, USA; xfu3@central.uh.edu (X.F.); xzhang5@central.uh.edu (S.X.Z.)
[3] MD Anderson Cancer Center, Department of Cancer Systems Imaging, 1881 East Road, Houston, TX 77030-4009, USA
* Correspondence: bguo@central.uh.edu (B.G.); gomika@uh.edu (D.G.U.)
† These authors contributed equally to this work.

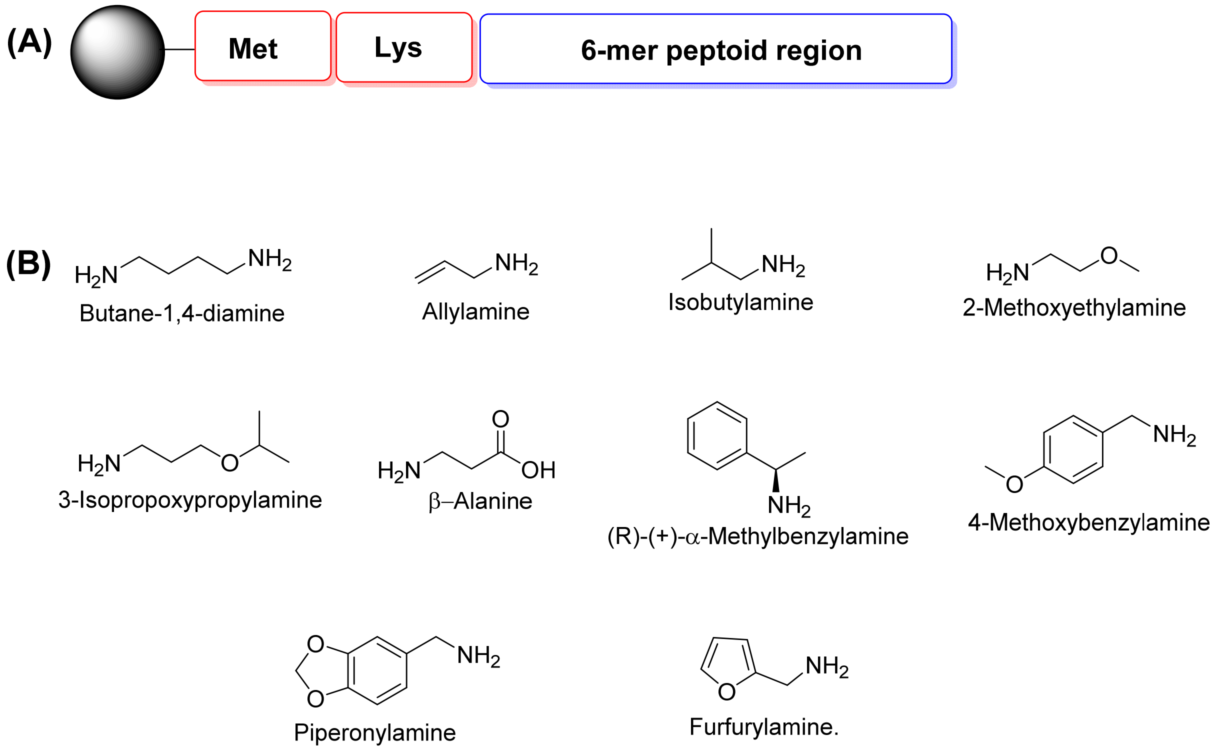

Supplementary Figure S1: (**A**). Outline of the peptoid library. (**B**). types of amines used in the peptoid library. Theoretical diversity of the library is 1,000,000

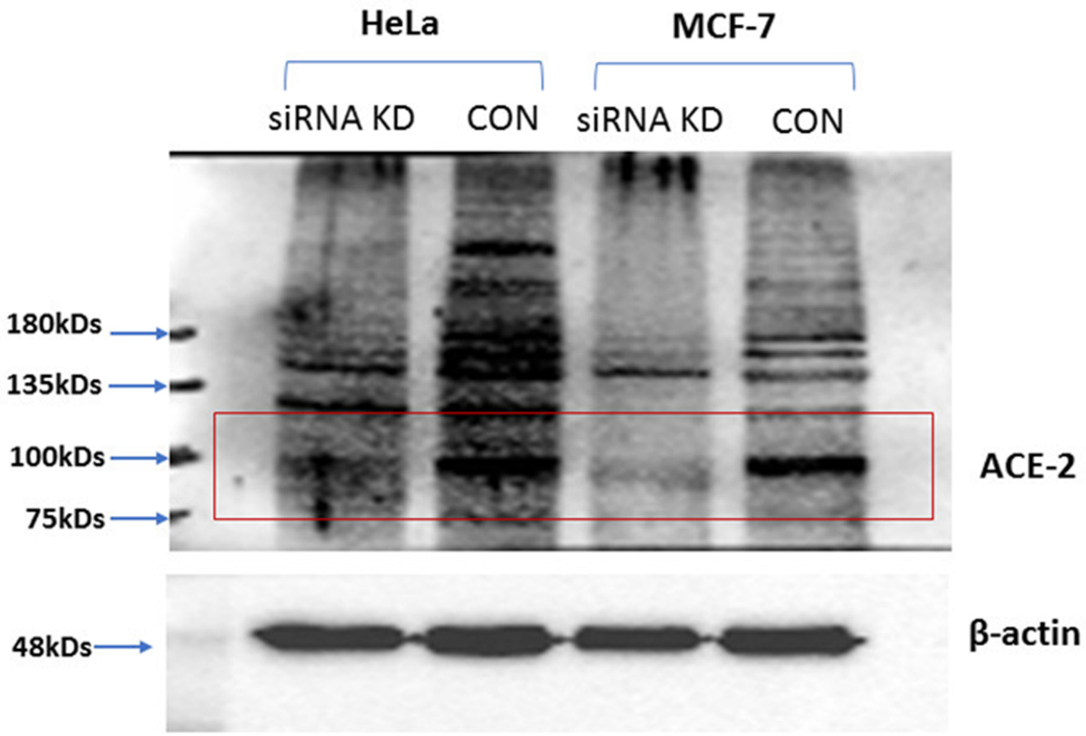

Supplementary Figure S2: MCF-7 and Hela cells express ACE2 and siRNA effectively decreases ACE2 expression. MCF-7 and HeLa cells were transfected with ACE2 targeting siRNA. ACE2 expression was detected by western blot and β-actin was used as loading control.

Note: Only MCF-7 cells were used for the OBTC assay.

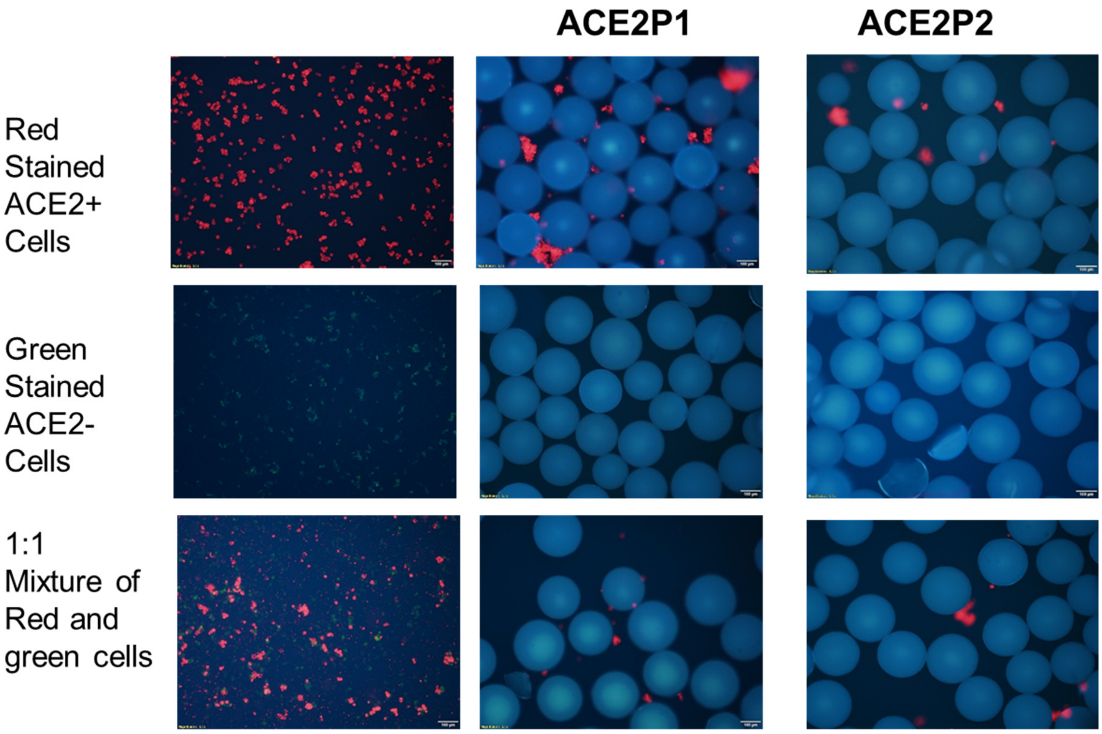

Supplementary Figure S3: On-bead peptoids ACE2P1 and ACE2P2 binds to human ACE2 positive cells. The compounds ACE2P1 and ACE2P2 were resynthesized on the tentagel beads and incubated with ACE2 expressing MCF-7 cells (red stained) and ACE2 negative MCF-7 cells (green stained) individually as well as 1:1 mixture of red and green cells. Beads only showed binding with red cells but not with green cells, indicating ACE2P1 and ACE2P2 binds to human ACE2 protein.

(A)

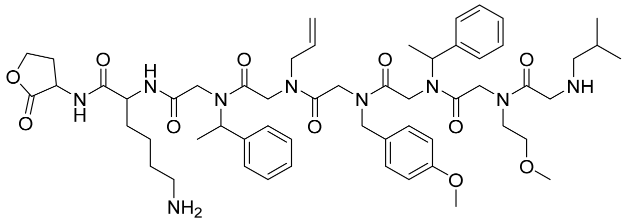
O
O
N
H
O
H
N
NH2
O
N
O
N
O
N
O
O
N
O
N
O
NH
O

(B)

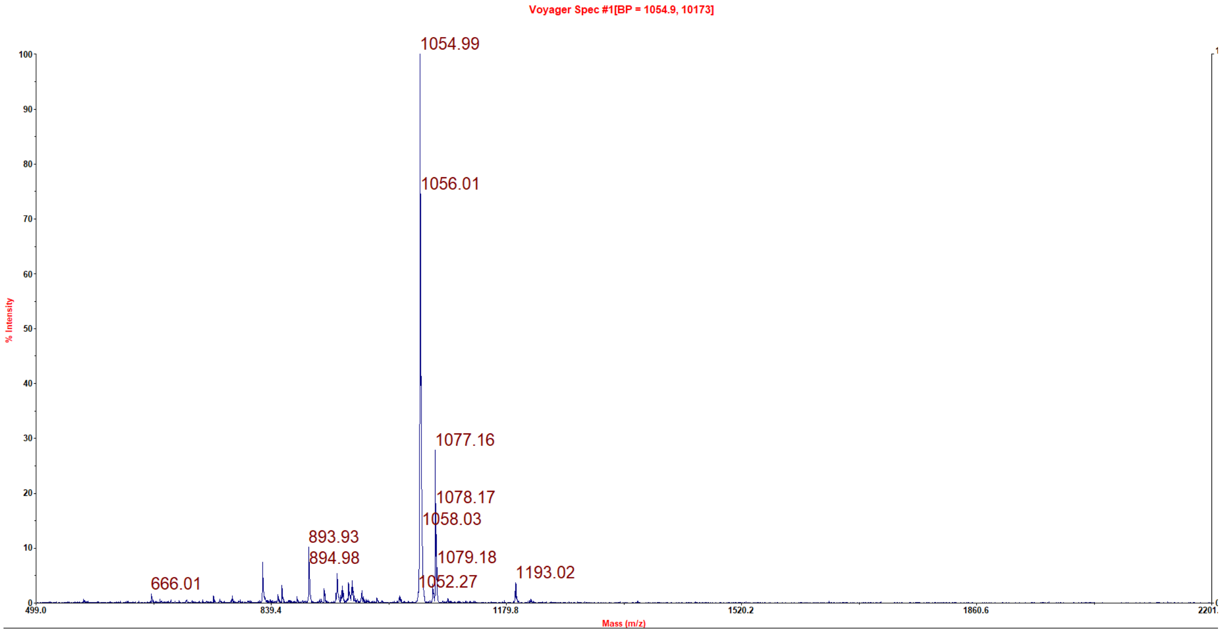
Voyager Spec #1[BP = 1054.9, 10173]
1054.99
1056.01
1077.16
1078.17
1058.03
893.93
894.98
1079.18
1193.02
666.01
1052.27
% Intensity
Mass (m/z)
499.0
839.4
1179.8
1520.2
1860.6
100
90
80
70
60
50
40
30
20
10
0

(C)

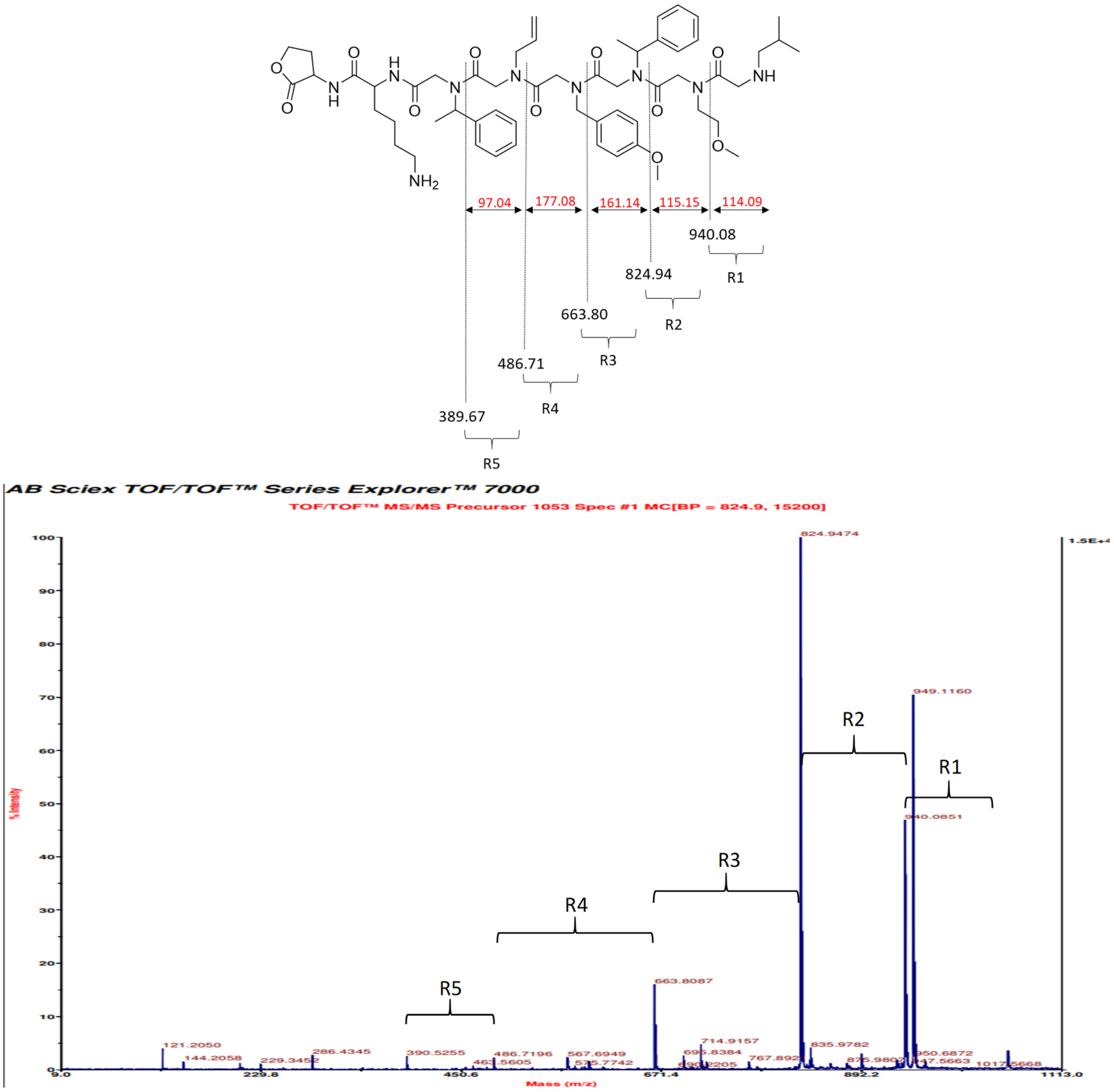

Supplementary Figure S4: Structure of ACE2P1 identified through MS/MS sequencing, synthesized on TentaGel MB NH_2 resin and cleaved by CNBr cleaving solution as described in Methods. (**A**) Structure of ACE2P1 identified through MS/MS sequencing, (**B**) MALDI-TOF data of ACE2P1, (**C**) MS/MS sequencing data of ACE2P1.

(A)

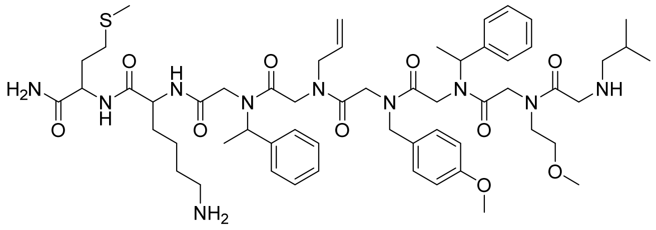

(B)

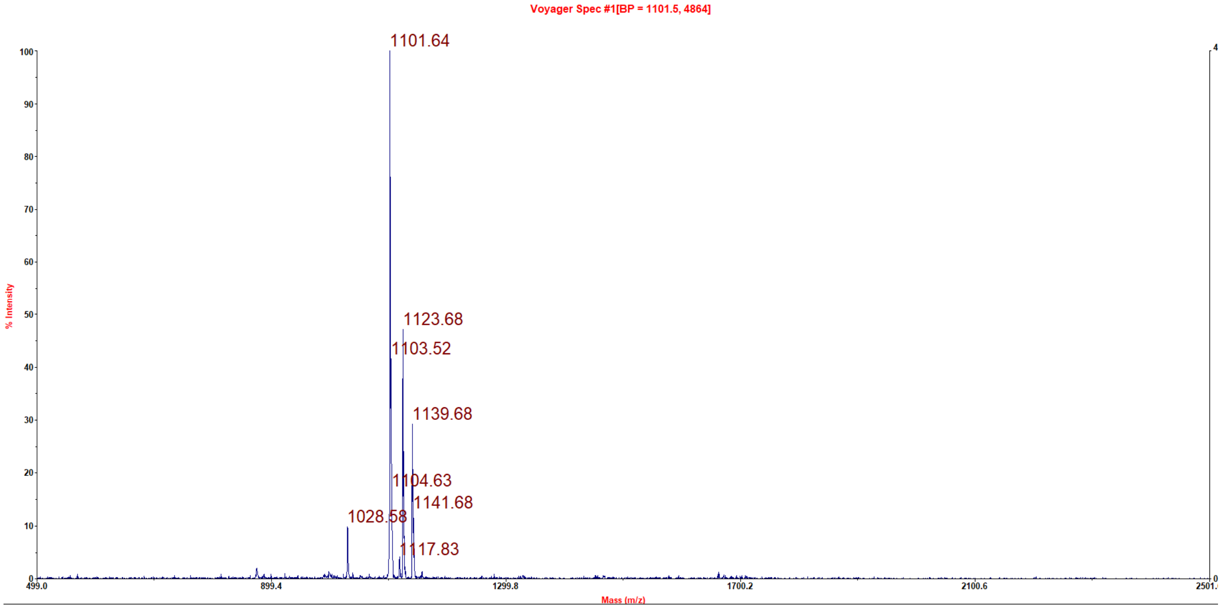

(C)

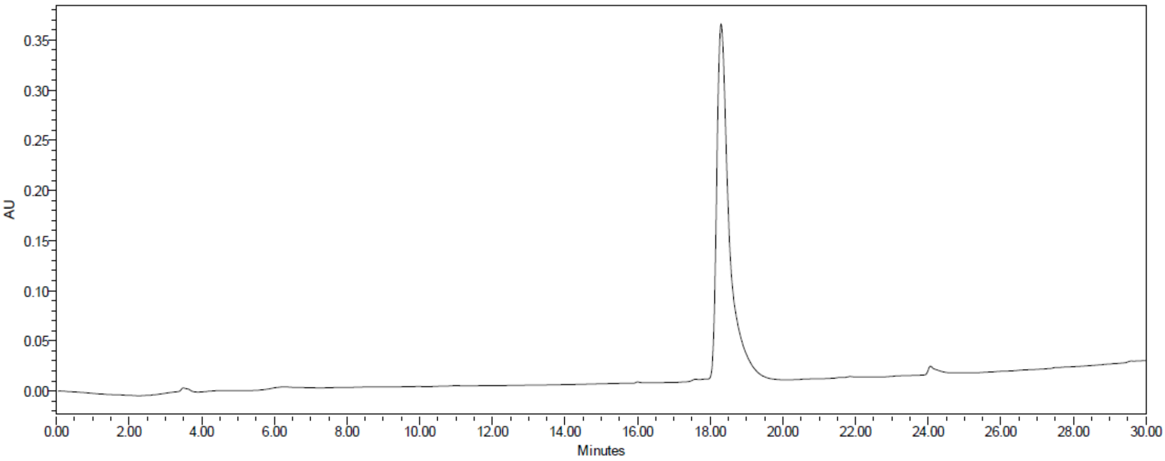

Supplementary Figure S5: Characterization of ACE2P1: (**A**) Chemical structure of ACE2P1, (**B**) MALDI-TOF spectrum of ACE2P1, (**C**) Analytical HPLC of ACE2P1.

(A)

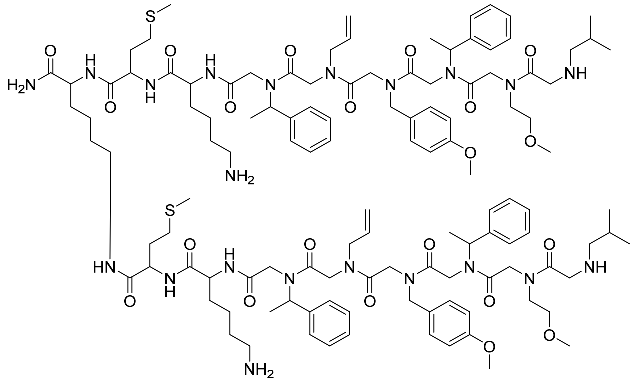

(B)

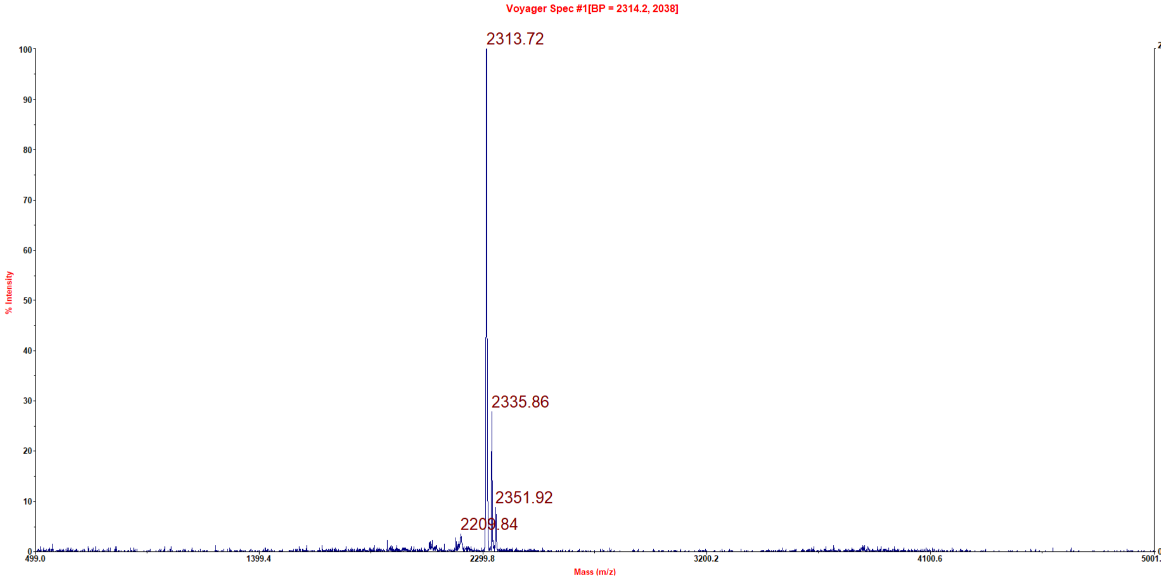

(C)

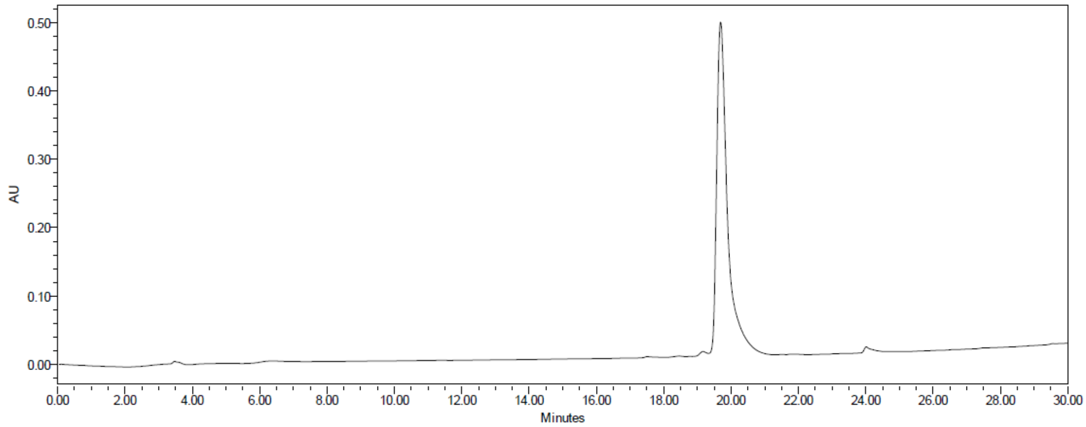

Supplementary Figure S6: Characterization of ACE2P1D1: (**A**) Chemical structure of ACE2P1D1, (**B**) MALDI-TOF spectrum of ACE2P1D1, (**C**) Analytical HPLC of ACE2P1D1.

(A)

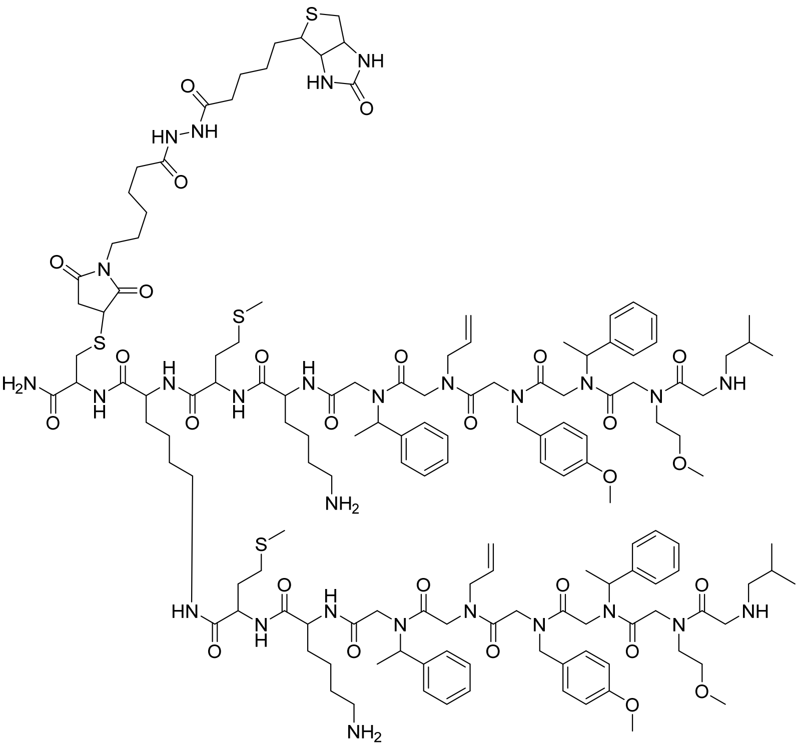

(B)

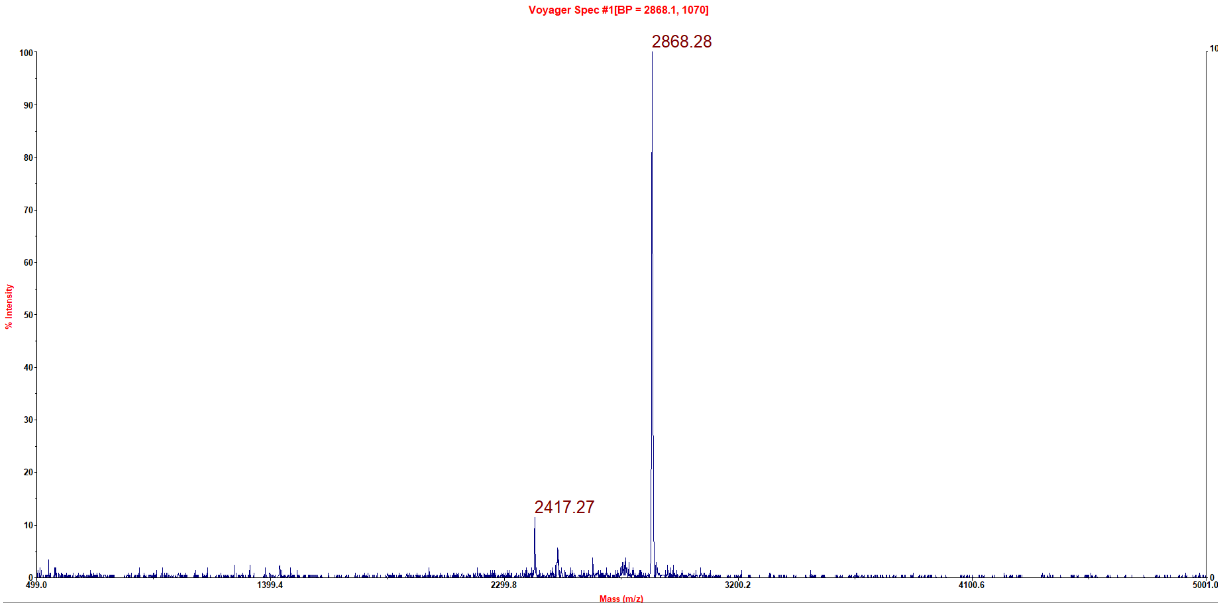
Voyager Spec #1[BP = 2868.1, 1070]
2868.28
2417.27
% Intensity
Mass (m/z)
499.0
1399.4
2299.8
3200.2
4100.6
5001.0

(C)

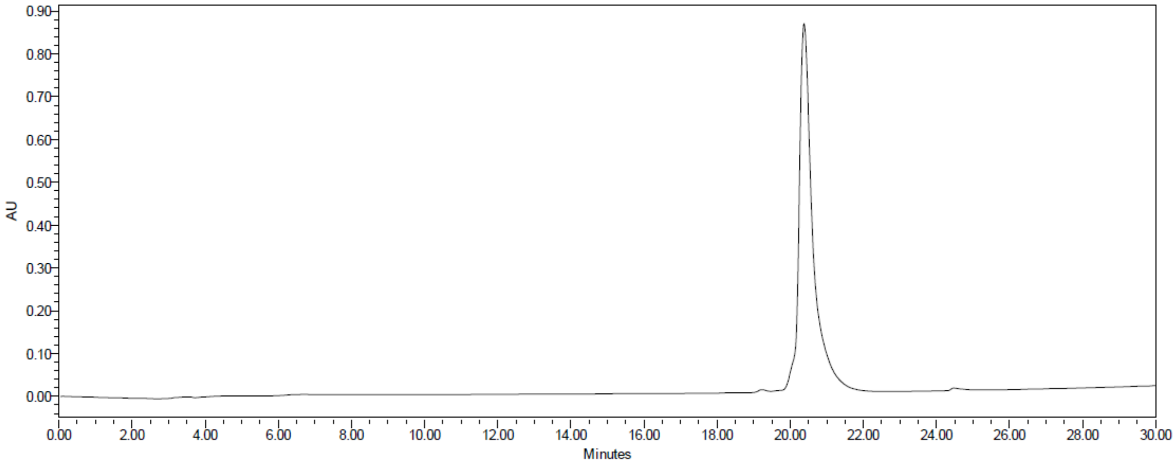

Supplementary Figure S7: Characterization of Biotin-ACE2P1D1**:** (**A**) Chemical structure of Biotin-ACE2P1D1, (**B**) MALDI-TOF spectrum of Biotin-ACE2P1D1, (**C**) Analytical HPLC of Biotin-ACE2P1D1.

(A)

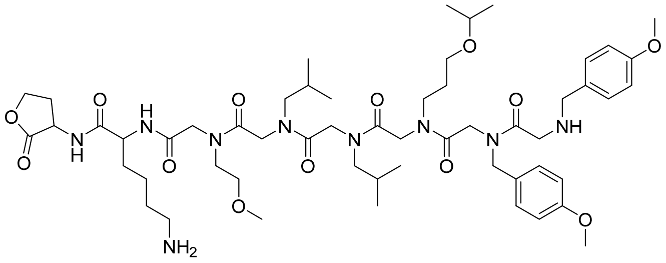

(B)

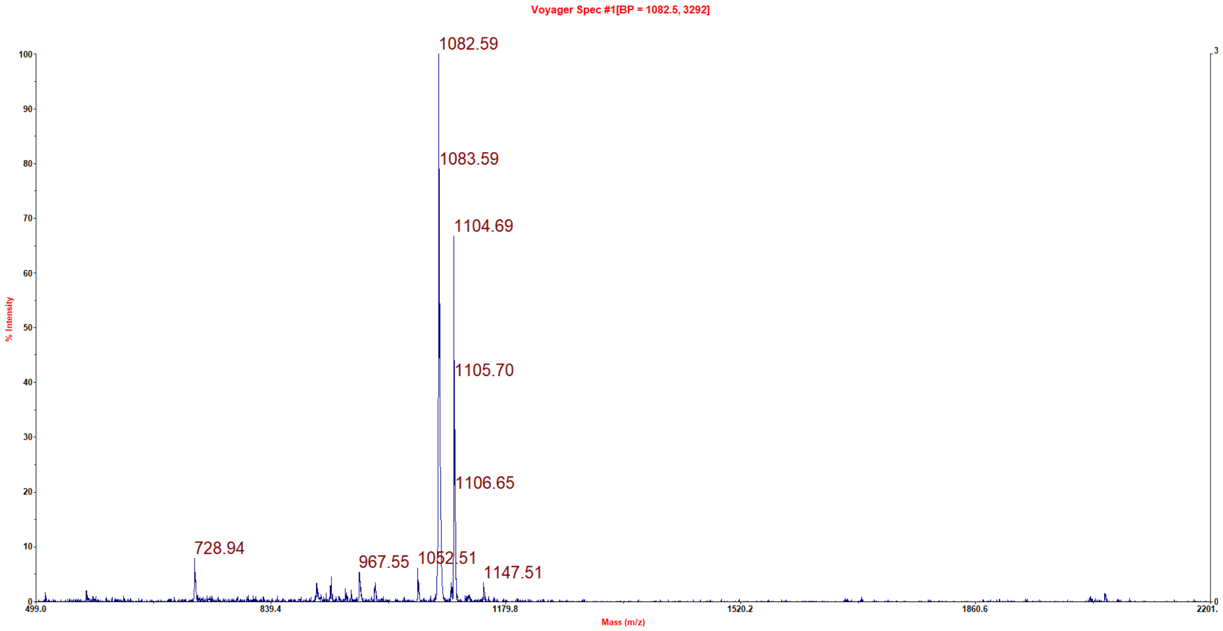
Voyager Spec #1[BP = 1082.5, 3292]
1082.59
1083.59
1104.69
1105.70
1106.65
728.94
967.55
1052.51
1147.51
% Intensity
Mass (m/z)
499.0
839.4
1179.8
1520.2
1860.6
2201.

(C)

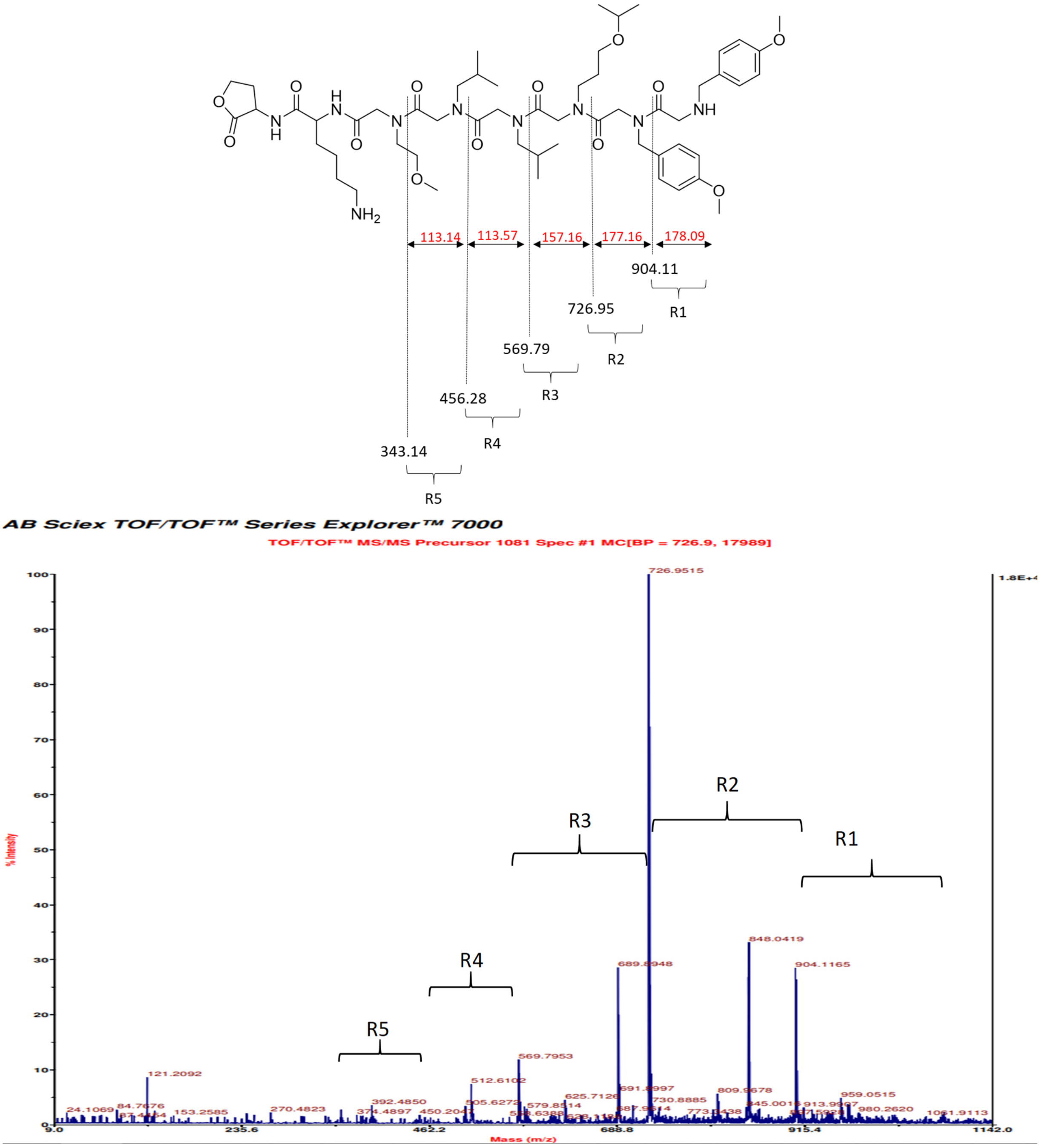

Supplementary Figure S8: Structure of ACE2P2 identified through MS/MS sequencing, synthesized on TentaGel MB NH_2 resin and cleaved by CNBr cleaving solution as described in Methods. (A) Structure of ACE2P2 identified through MS/MS sequencing, (B) MALDI-TOF data of ACE2P2, (C) MS/MS sequencing data of ACE2P2.

(A)

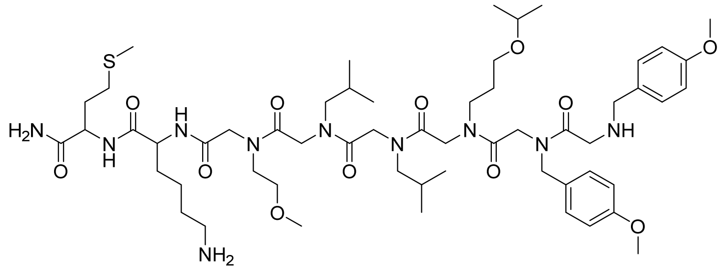

(B)

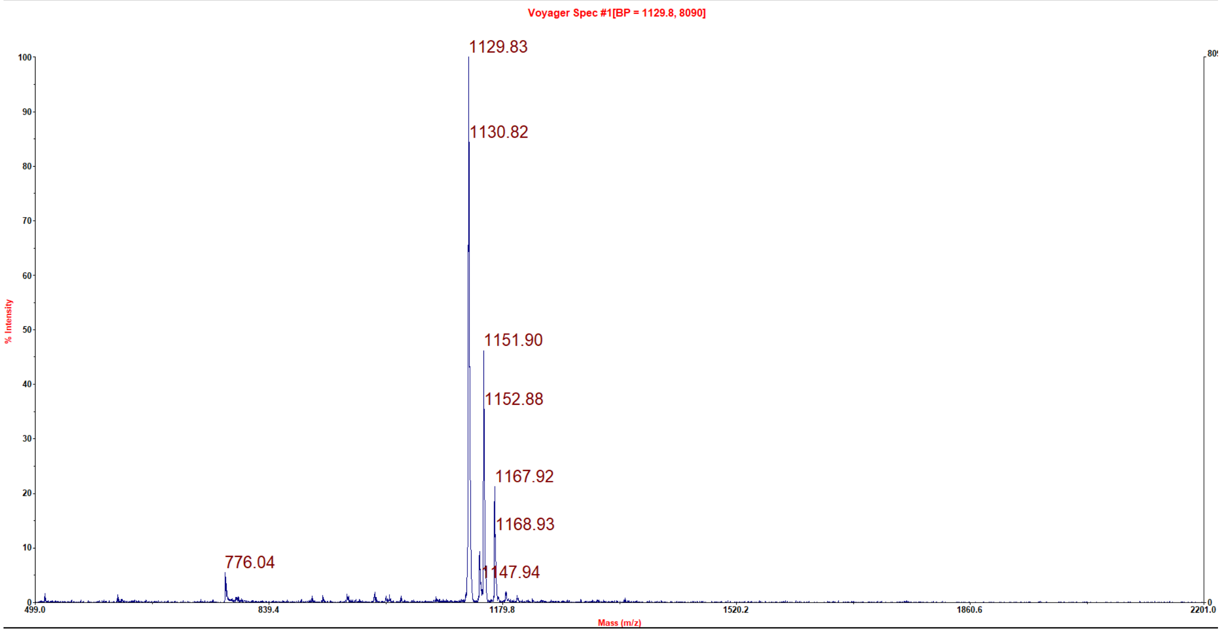

(C)

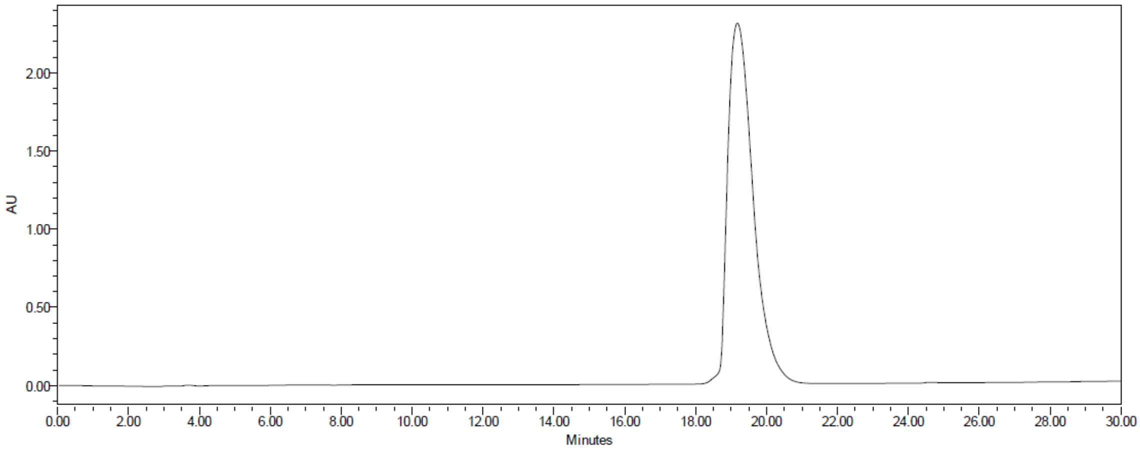

Supplementary Figure S9: Characterization of ACE2P2: (**A**) Chemical structure of ACE2P2, (**B**) MALDI-TOF spectrum of ACE2P2, (**C**) Analytical HPLC of ACE2P2.

(A)

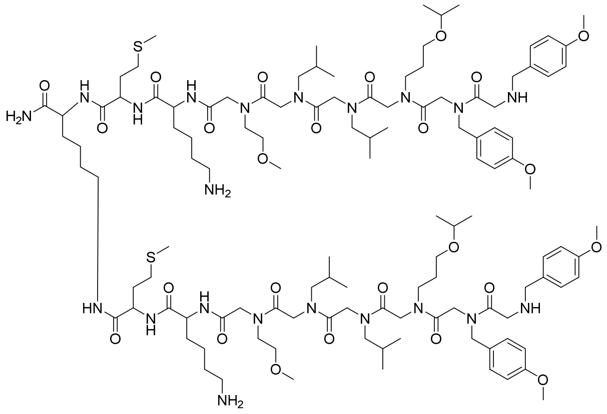

(B)

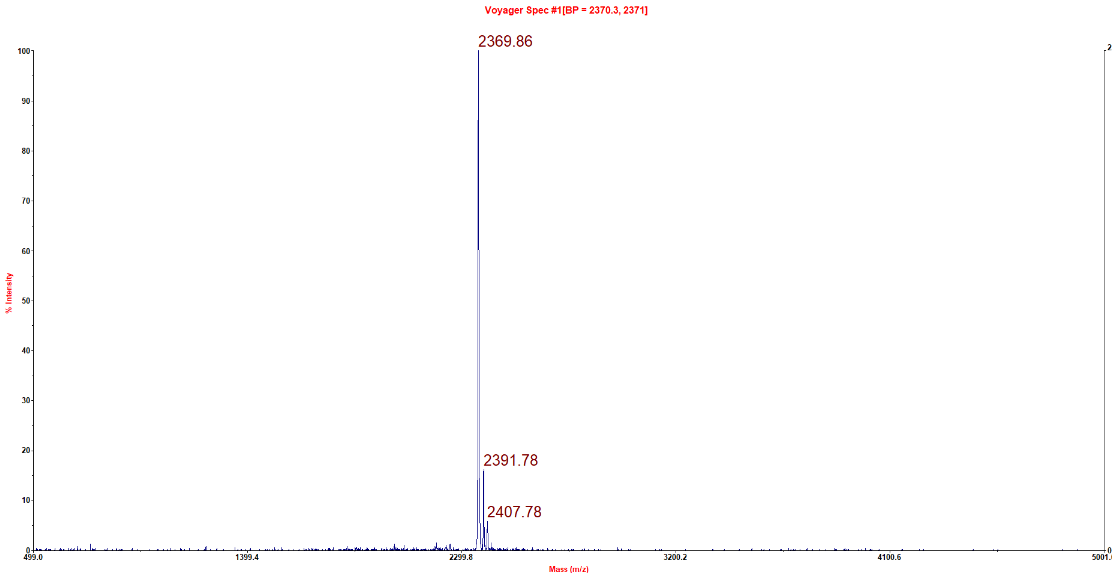

(C)

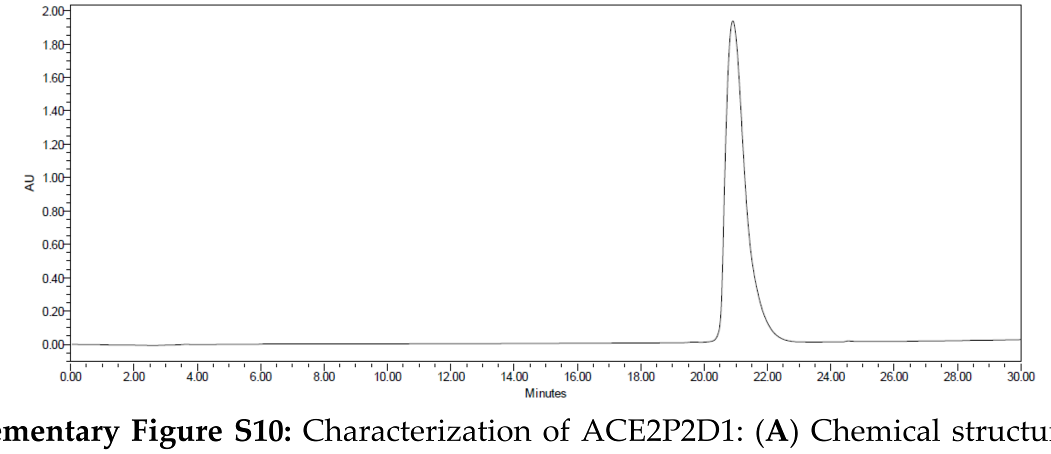

Supplementary Figure S10: Characterization of ACE2P2D1: (**A**) Chemical structure of ACE2P2D1, (**B**) MALDI-TOF spectrum of ACE2P2D1, (**C**) Analytical HPLC of ACE2P2D1.

(A)

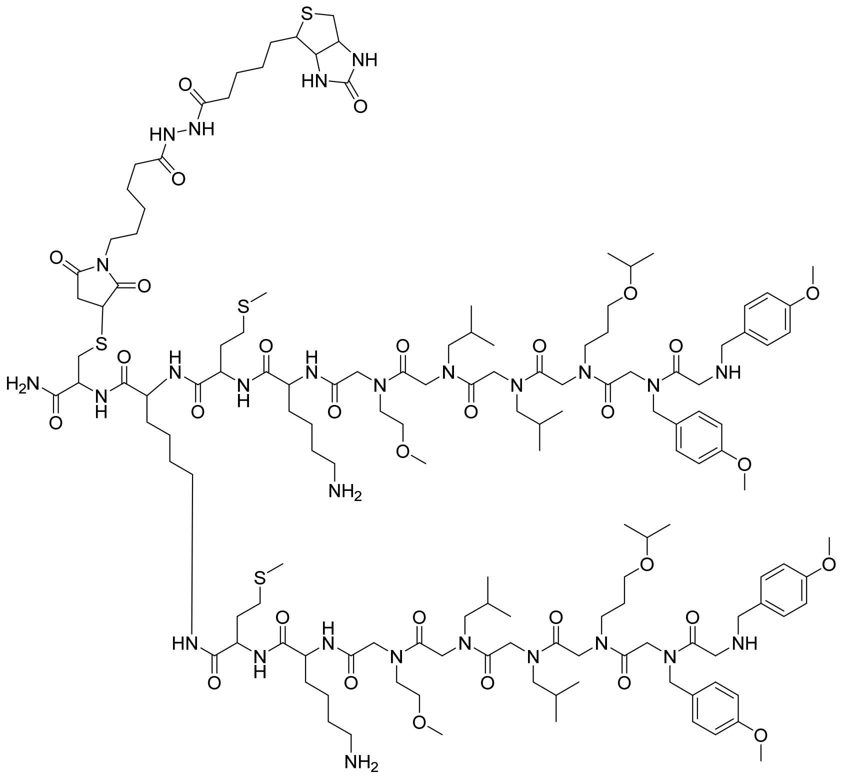

(B)

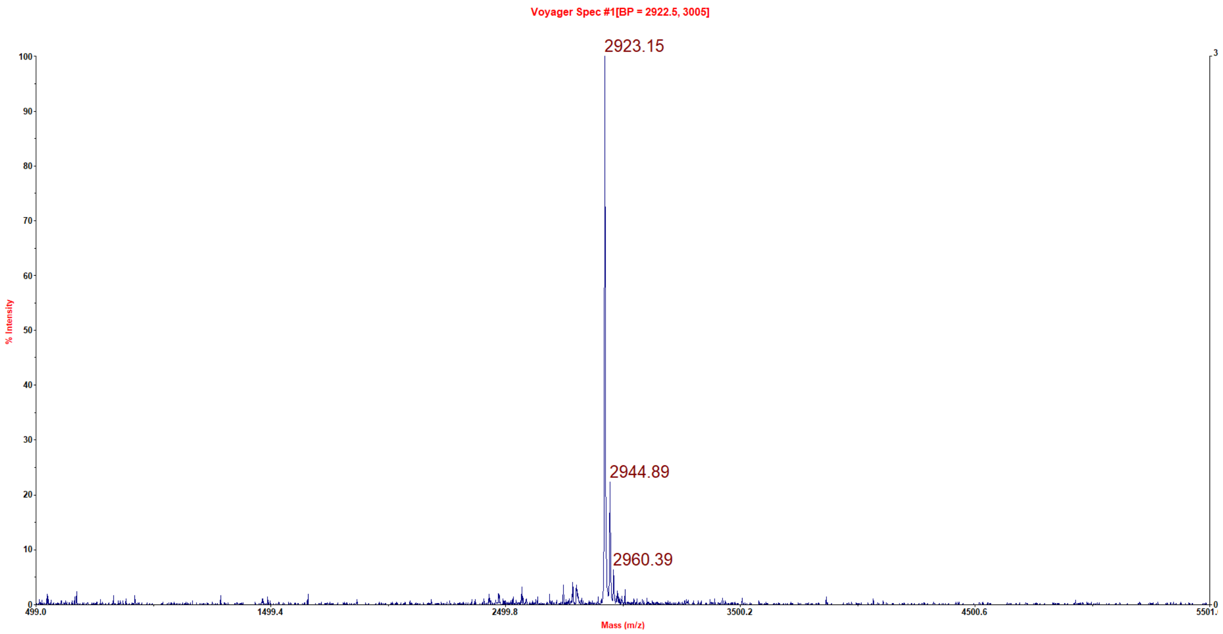
Voyager Spec #1[BP = 2922.5, 3005]
2923.15
2944.89
2960.39
% Intensity
Mass (m/z)
499.0
1499.4
2499.8
3500.2
4500.6
5501.

(C)

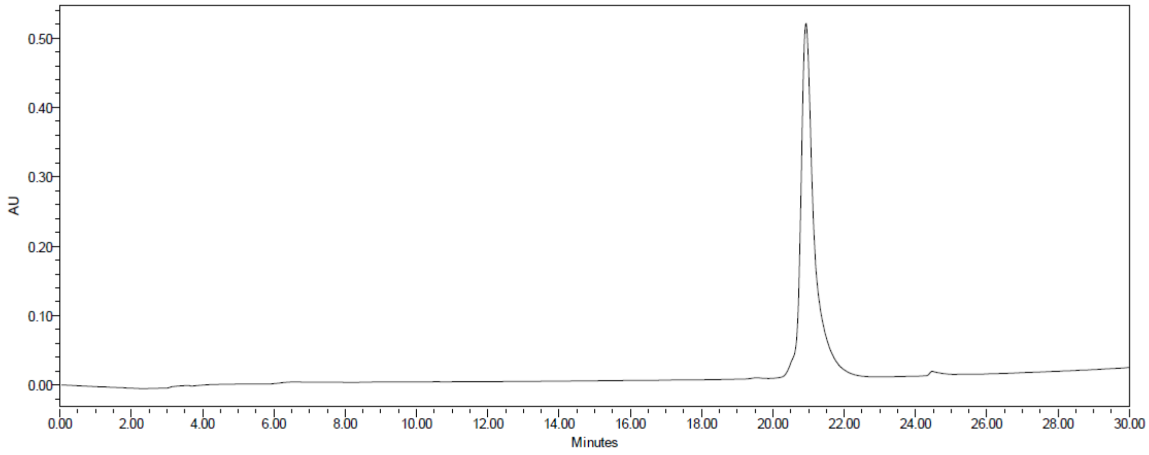

Supplementary Figure S11: Characterization of Biotin-ACE2P2D1: (**A**) Chemical structure of Biotin-ACE2P2D1, (**B**) MALDI-TOF spectrum of Biotin-ACE2P2D1, (**C**) Analytical HPLC of Biotin-ACE2P2D1.

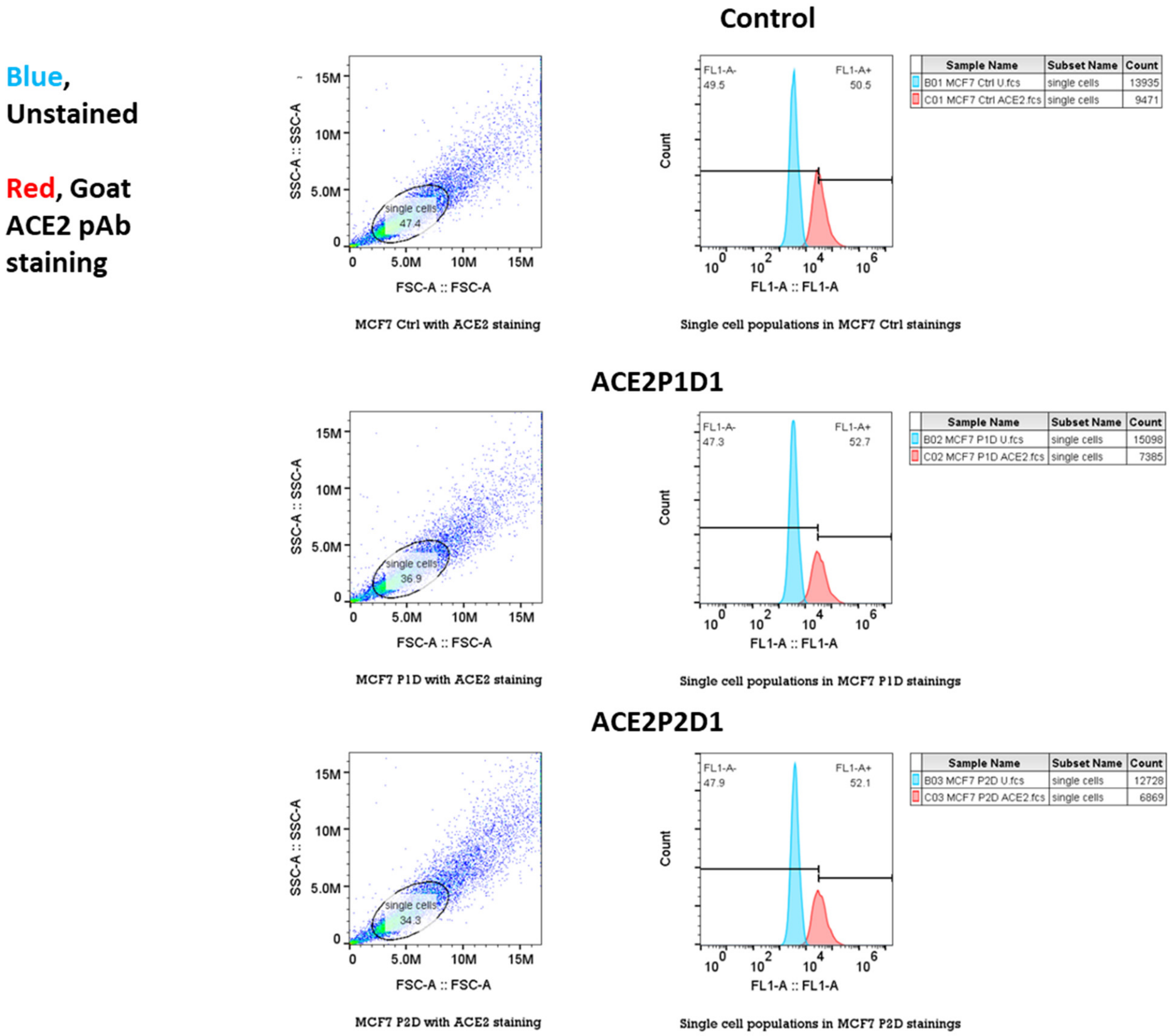

Supplementary Figure S12: ACE2P1D1 and ACE2P2D1 do not decrease the expression levels of human ACE2**.** ACE2P1D1 and ACE2P2D1 do not decrease ACE2 expression on cell surfaces. MCF-7 cells were treated with 10 μM of ACE2P1D1 and ACE2P2D1 for 48 hours. ACE2 surface expression was determined by flow cytometry analysis.

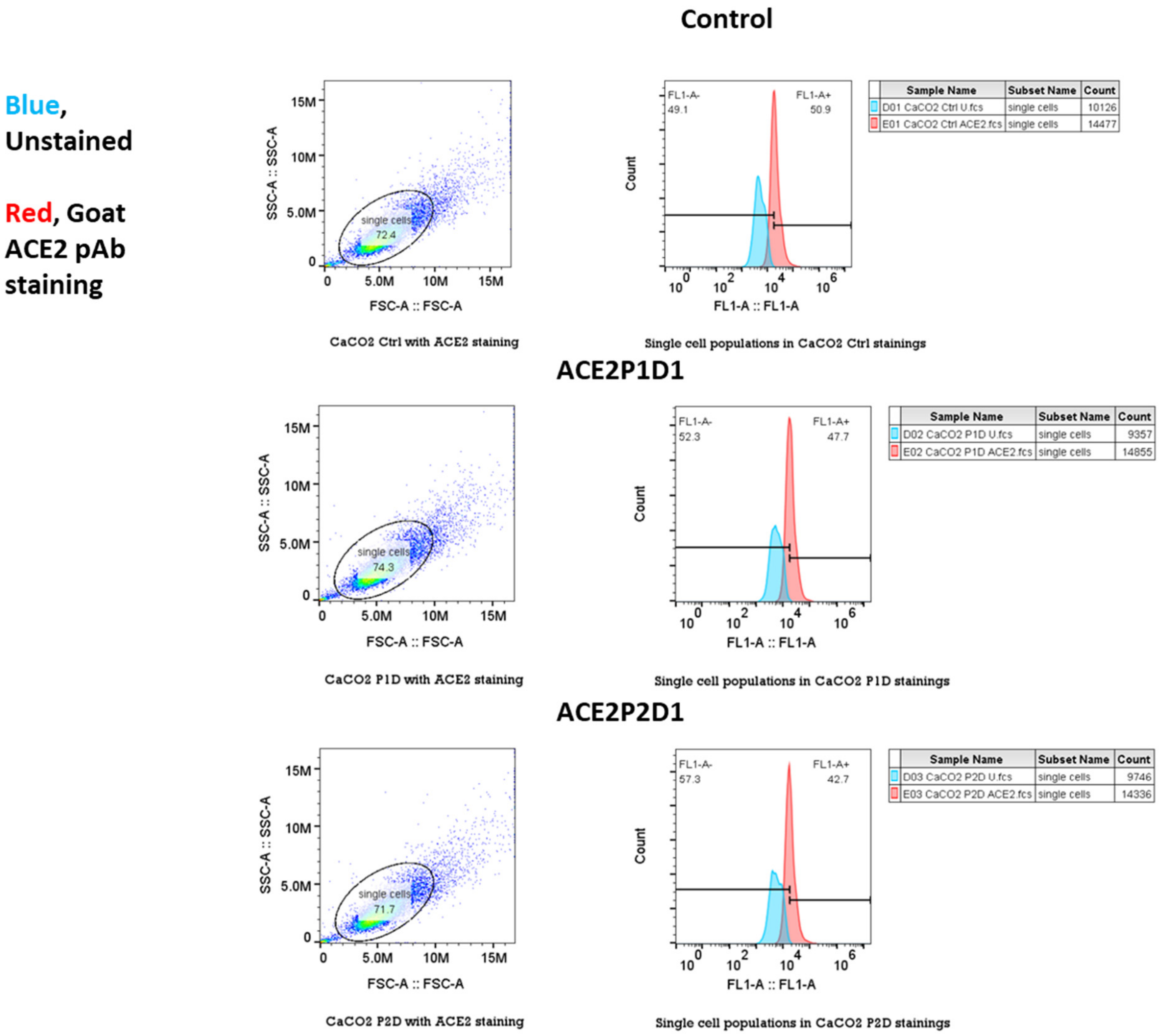

Supplementary Figure S13: ACE2P1D1 and ACE2P2D1 do not decrease the expression levels of human ACE2. ACE2P1D1 and ACE2P2D1 do not decrease ACE2 expression on cell surfaces. Caco-2 cells were treated with 10 μM of ACE2P1D1 and ACE2P2D1 for 48 hours. ACE2 surface expression was determined by flow cytometry analysis.

Original data:

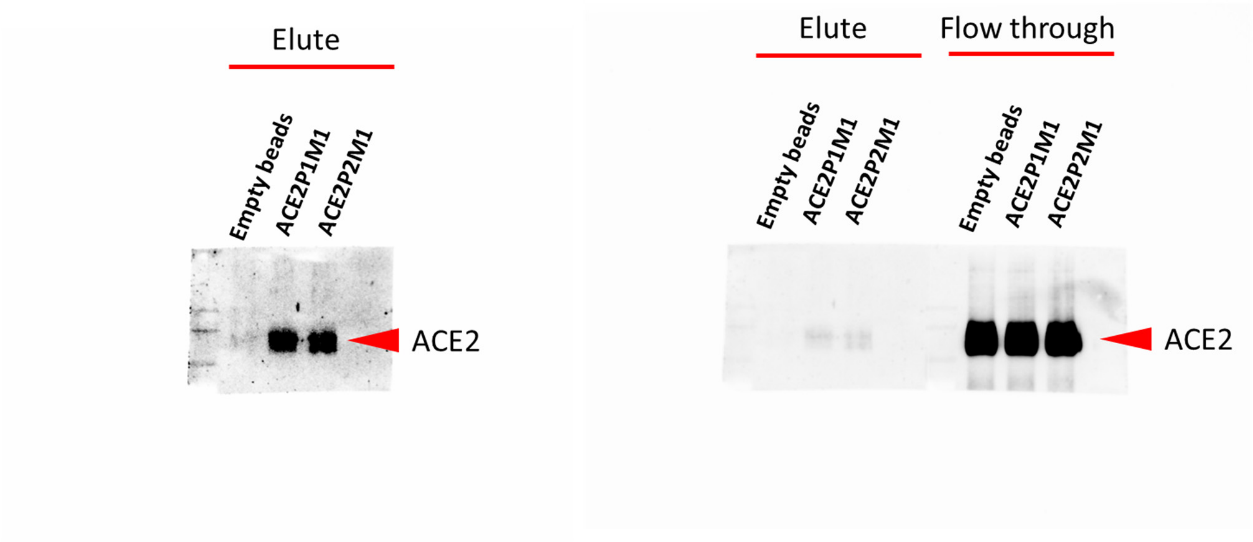

Supplementary Figure S14: The original western blots for Figure 1g. OBTC peptoid screen identifies two potential human ACE2 binding peptoids. TentaGel beads carrying ACE2P1 and ACE2P2 peptoids or empty beads (as control) were incubated with recombinant ACE2 protein. The beads were washed thoroughly and protein was eluted and analyzed by western blot. Blots were cropped from different parts of the same gel.

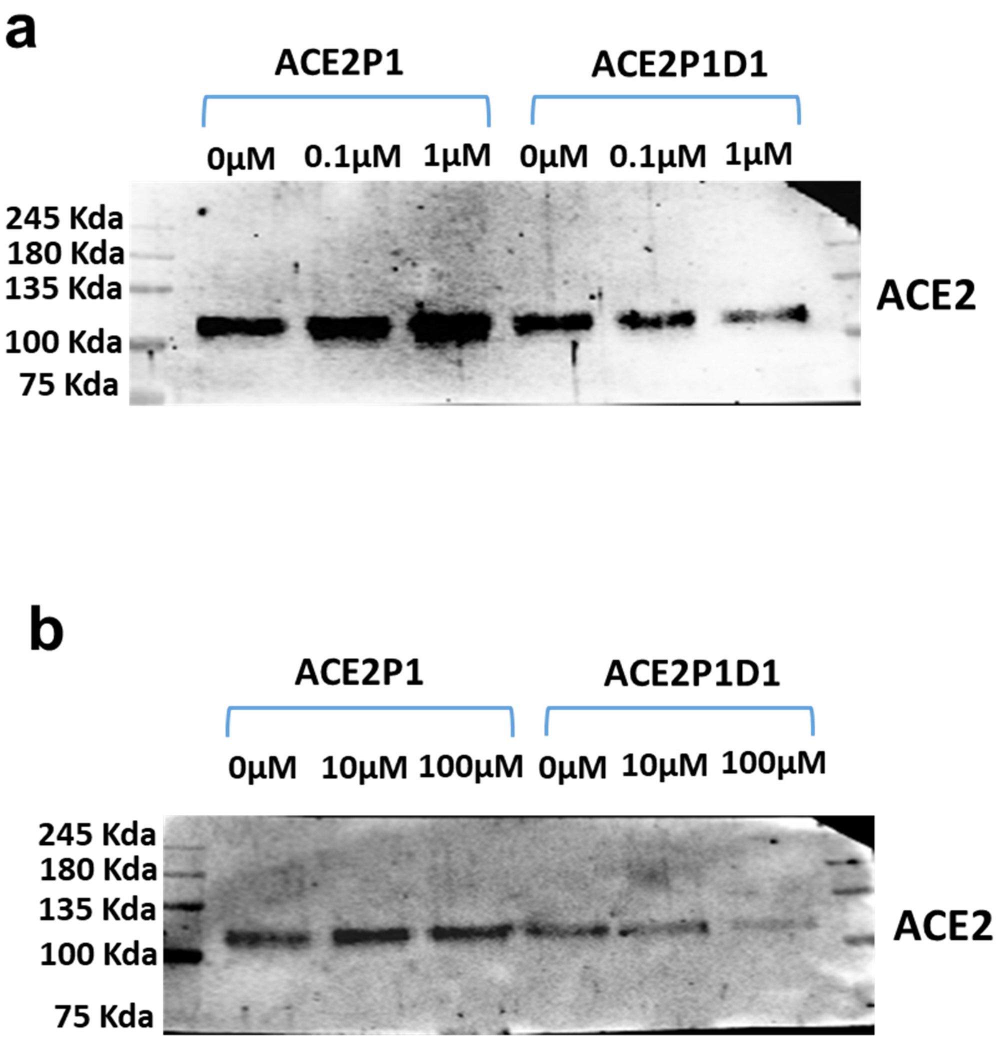
a
ACE2P1
ACE2P1D1
0μM
0.1μM
1μM
0μM
0.1μM
1μM
245 Kda
180 Kda
135 Kda
100 Kda
75 Kda
ACE2
b
ACE2P1
ACE2P1D1
0μM
10μM
100μM
0μM
10μM
100μM
245 Kda
180 Kda
135 Kda
100 Kda
75 Kda
ACE2

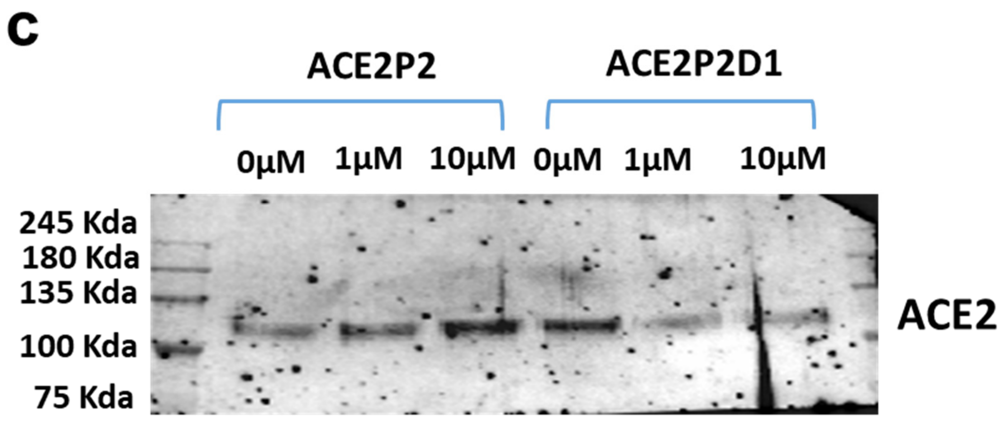

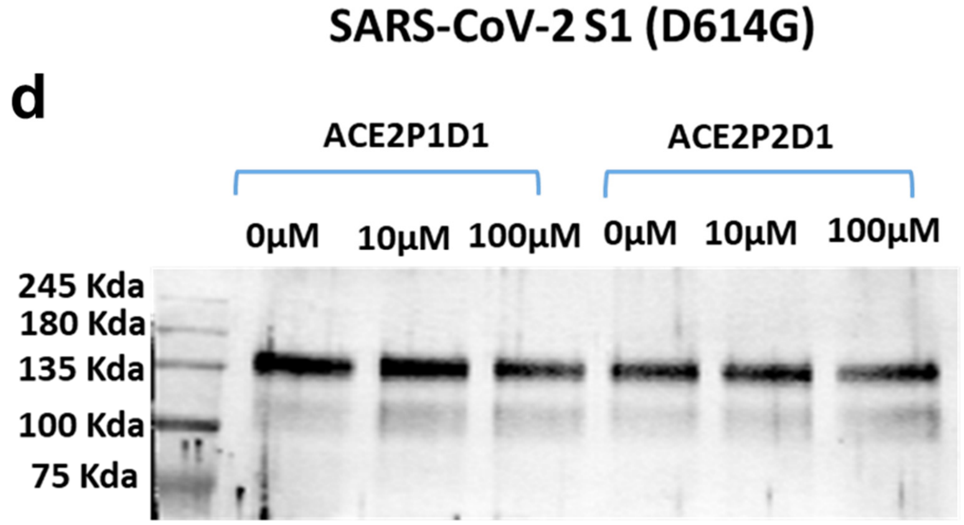

Supplementary Figure S15: The original western blots for Figure 2. ACE2P1 and ACE2P2 dimers blocked the binding of spike protein to ACE2. **(a-c)** Recombinant ACE2 (0.1µg/sample) and recombinant GST-tagged SARS-CoV2 spike proteins (0.1µg/sample) were mixed together. ACE2P1 and ACE2P2 monomers or dimers (D1) were premixed with ACE2 for 1 hour before the mixture was applied to the GST pull-down assay to determine if these compounds can block the interaction between ACE2 and spike protein. (**d**) The interaction of recombinant ACE2 and D614G

spike proteins (0.1 μg/sample) was determined by pull-down assay in the presence or absence of various concentrations of the peptoids.

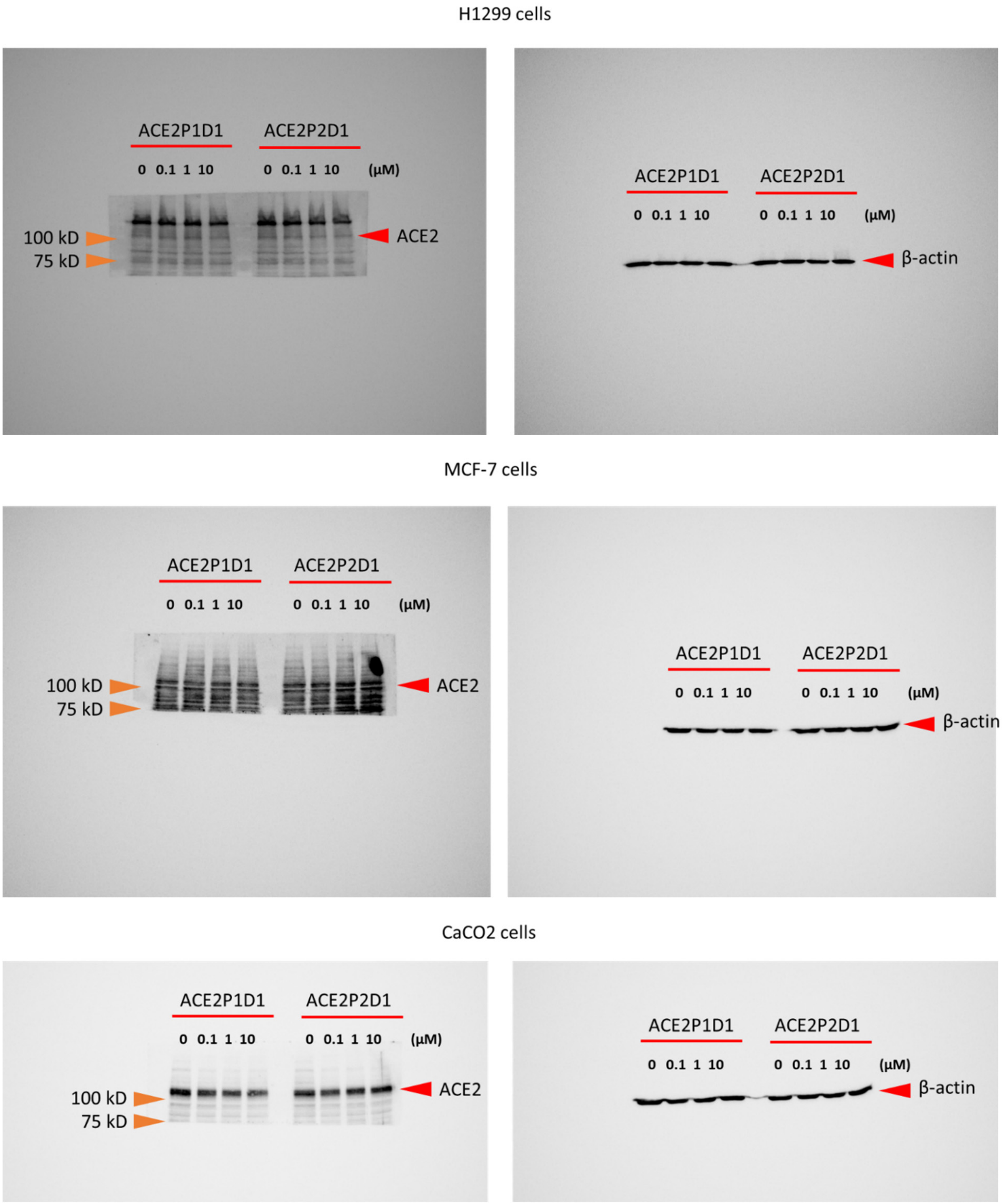

Supplementary Figure S16: The original western blots for Figure 5b. ACE2P1D1 and ACE2P2D1 do not decrease ACE2 expression and are not toxic to cells. H1299, MCF-7, and Caco-2 cells were treated with various concentrations of ACE2P1D1 or ACE2P2D1 for 48 hours. Total ACE2 levels in cells were analyzed by western blots.
